# Morphological Innovation Drives Sperm Release in Bryophytes

**DOI:** 10.1002/advs.202306767

**Published:** 2024-03-29

**Authors:** Xinxin Zhang, Ang Bian, Junbo Yang, Ye Liang, Zhe Zhang, Meng Yan, Siqi Yuan, Qun Zhang

**Affiliations:** ^1^ Institute of Botany Chinese Academy of Sciences Beijing 100093 China; ^2^ College of Computer Science Sichuan University Chengdu 610065 China; ^3^ Shenzhen Branch Guangdong Laboratory of Lingnan Modern Agriculture Genome Analysis Laboratory of the Ministry of Agriculture and Rural Affairs Agricultural Genomics Institute at Shenzhen Chinese Academy of Agricultural Sciences Shenzhen Guangdong 518120 China; ^4^ Core Facility of the State Key Laboratory of Membrane Biology Peking University Beijing 100871 China; ^5^ Department of Mechanical and Aerospace Engineering The Hong Kong University of Science and Technology Clear Water Bay Hong Kong 999077 China; ^6^ School of Life Science Hangzhou Institute for Advanced Study University of Chinese Academy of Sciences Hangzhou Zhejiang 310024 China; ^7^ College of Life Sciences State Key Laboratory of Crop Genetics & Germplasm Enhancement and Utilization Nanjing Agricultural University Nanjing 210095 China

**Keywords:** antheridium burst, cell geometry, cell wall mechanics, hydrostatic pressure, *Marchantia polymorpha*, *Physcomitrium patens*, sperm release

## Abstract

Plant movements for survival are nontrivial. Antheridia in the moss *Physcomitrium patens* (*P. patens*) use motion to eject sperm in the presence of water. However, the biological and mechanical mechanisms that actuate the process are unknown. Here, the burst of the antheridium of *P. patens*, triggered by water, results from elastic instability and is determined by an asymmetric change in cell geometry. The tension generated in jacket cell walls of antheridium arises from turgor pressure, and is further promoted when the inner walls of apex burst in hydration, causing water and cellular contents of apex quickly influx into sperm chamber. The outer walls of the jacket cells are strengthened by NAC transcription factor VNS4 and serve as key morphomechanical innovations to store hydrostatic energy in a confined space in *P. patens*. However, the antheridium in liverwort *Marchantia polymorpha* (*M. polymorpha*) adopts a different strategy for sperm release; like jacket cell outer walls of *P. patens*, the cells surrounding the antheridium of *M. polymorpha* appear to play a similar role in the storage of energy. Collectively, the work shows that plants have evolved different ingenious devices for sperm discharge and that morphological innovations can differ.

## Graphical abstract

The biomechanics of antheridium burst in moss *Physcomitrium patens* depend on a single layer of jacket cells that store hydrostatic energy, which results from elastic instability and asymmetric changes in cell geometry. By contrast, the antheridium in liverwort *Marchantia polymorpha* adopts a different strategy for sperm release, and the cells surrounding the antheridium appear to play a dominant role.

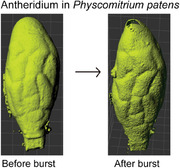

## 1 Introduction

Movements in plants are relatively rare but fulfill essential functions that ensure the survival of the species. For example, some plants have adapted rapid movements for seed, pollen, and spore dispersal,^[^ ^1^ ^]^ and for catching insects.^[^ ^2^ ^]^ The generation of most rapid movements highly relies on elastic instabilities that can be primarily classified into two categories: snap‐buckling and explosive fracture, which involves rapid geometric changes.^[^ ^3^ ^]^ During the snap of a Venus fly trap, for example, leaf geometry changes from convex to concave.^[^ ^2a^ ^]^ In *Cardamine hirsuta*, the asymmetrically lignified secondary cell wall in the endocarp *b* cell layer of the fruit valve widens during seed pod explosion.^[^ ^1a^ ^]^ In fern sporangia, the strong change in the curvature of the whole annulus forces the opening of the sporangium at the stomium, leading to the expulsion of the spores.^[^ ^1b^ ^]^ Thesechanges in geometry lead to a rapid release of the elastic energy that is gradually stored.^[^ ^1^, ^2^, ^3^ ^]^ In certain explosive fruits, storage of elastic energy relies on hydrostatic pressure. For example, squirting cucumber (*Ecballium elaterium*) and dwarf mistletoes (*Arceuthobium spp*.) accumulate considerable hydrostatic pressure in their fruits until they ultimately explode.^[^ ^1^, ^4^ ^]^

Water‐mediated fertilization has been lost in most seed plants but is a common characteristic in early diverging land plants, including bryophytes and ferns,^[^ ^5^ ^]^ which has evolved different strategies to expose flagellated sperms that swim to a stationary egg.^[^ ^6^ ^]^ In contrast to dehydration‐driven anther dehiscence in seed plants, the antheridium burst is water‐driven to release mature spermatozoids in the moss species.^[^ ^5^, ^7^ ^]^ This phenomenon has been widely reported for decades, but the detailed process of antheridium dehiscence has not been described, and the biological and mechanical mechanisms involved in sperm release remain unknown. Here we showed sperm release from the antheridium of moss model species *Physcomitrium* *patens* (*P. patens*) and presented a mechanism to generate tension and release it.

## 2 Results and Discussion

### 2.1 Changes of Jacket Cell Geometry Trigger Rapid Sperm Release

**Figure 1:**
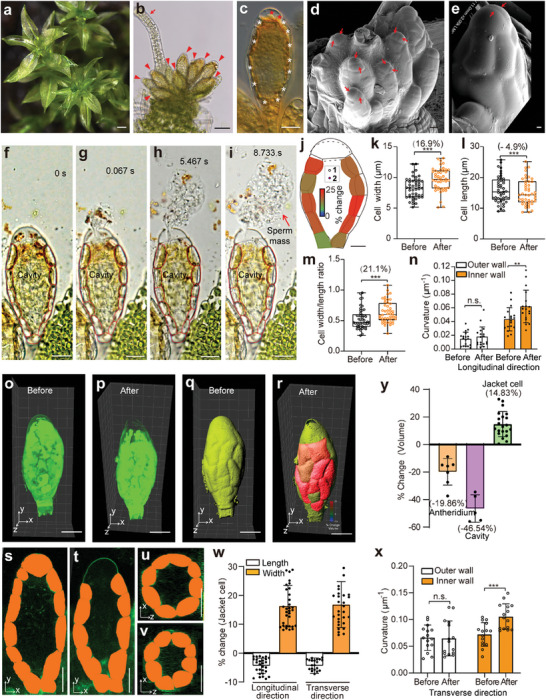
Jacket cell geometry changed after antheridium burst in *P. patens*. a) Gametophores. b) Gametophore apices with antheridia and archegonia. Arrowheads indicate antheridia. Arrow indicates archegonia. c) Individual antheridium with a single layer of jacket cells. White and red asterisks indicate jacket and apical cells, respectively. d) Cryo‐SEM image of antheridia. Red arrows indicate apical cells. The apex is generally composed of two apical cells. e) Close view of antheridium apex. Red arrows indicate apical cells. f–i) The antheridium geometry before f) and after burst g–i). Red dashed lines indicate jacket cells. j) Simulated changes of a mature antheridium with jacket cells before and after burst. Five antheridia are computed. Number 1 and 2 represent the antheridium corresponding to before and after burst, respectively. Dashed lines indicate ruptured apex walls. Heat‐map indicates the area extension of the jacket cell. k–m) Quantitative analysis of jacket cell width k), length l) and cell width/length ratio m) before and after antheridium burst in the longitudinal direction. Box plots depict min to max values. Results are mean ± s.d. (*n* ≥ 45); two‐tailed paired Student's *t*‐test; ***P < 0.001. n) Quantification of curvature of jacket cell walls before and after antheridium burst in the longitudinal direction. Results are mean ± s.d. (*n* = 18); two‐way ANOVA with Dunnett's test; **P < 0.01, n.s., no significance (*P* > 0.05). o,p) 3D fluorescence images of the same antheridium before o) and after p) burst. q,r) 3D models of the same antheridium before q) and after r) burst reconstructed based on the fluorescence from o) and p), respectively. Heat‐maps indicate jacket cell volume extension after burst in r). s,t) Jacket cells from the same antheridium in the X‐Y plane before s) and after t) antheridium burst. u,v) Jacket cells from the same antheridium in the X‐Z plane before u) and after v) burst. The jacket cells have been false‐shaded in orange for clarity s–v). w) Quantification of the change in length and width of the jacket cell in the longitudinal and transverse directions after the antheridium burst, measured as the percent change. Results are mean ± s.d. (*n* ≥ 21). x) Quantification of curvature of jacket cell walls before and after antheridium burst in the transverse direction. Results are mean ± s.d. (*n* = 15); two‐way ANOVA with Dunnett's test; ***P < 0.001, n.s., no significance (*P* > 0.05). y) Quantification of the volume changes of the antheridium, cavity, and jacket cell after the antheridium burst, measured as the percent change. Results are mean ± s.d. (*n* ≥ 5). Scale bars, 1 mm in a); 50 µm in b); 20 µm in c, f‐i, o‐r, s‐v); 10 µm in d, j); 2 µm in e).

In *P. patens*, the antheridia are formed at the apex of the gametophore under low temperatures and short‐day conditions (**Figure** **1**a,b; Figure S1a–c, Supporting Information).^[^ ^8^ ^]^ The capsule of each antheridium in *P. patens* exclusively consists of a single layer of sterile jacket cells with two apical cells that form an apex, which surrounds a mass of spermatocytes (Figure 1c–e). Here, we first recorded sperm release of *P. patens* in 2D using high‐speed videography (Movie S1, Supporting Information). When a mature antheridium was submerged in water, a sperm mass consisting of biflagellated spermatozoids was released through a rupture at the top of the antheridium (Figure 1f–i). We also tracked the released speed of individual sperm cells in excess of 400 µm s^−1^ (Figure S1d–f; Movie S1, Supporting Information). The antheridium took about less than 9 s to completely empty their contents (Figure S1g; Movie S1, Supporting Information). Similar to the sperm mass release in *Bryum argenteum*,^[^ ^9^ ^]^ the sperm mass had larger area than the cavity as it was released from antheridium in *P. patens* (Figure 1i; Figure S1h, Supporting Information). The sperm cells in the sperm mass started rotating aimlessly in irregular circles a few seconds after release (Movie S2, Supporting Information). Subsequently, the sperm cells can leave the sperm mass and freely swim by vibrating its long flagella (Movie S3, Supporting Information). Moreover, the antheridium was contracted after sperm release, with antheridium area and width in longitudinal direction reduced by ≈9.4% and 5.0%, respectively, and the cavity area reduced by ≈21.9% (Figure 1f–j; Figure S1i–k, Supporting Information).

We next reconstructed the dynamics of antheridium geometry of *P. patens* during burst in 2D (Figure 1j) and observed the deformed shape of individual jacket cells therein (Figure 1f–n). Jacket cell inner walls largely extended toward the cavity after antheridium burst, with increased curvature (Figure 1f–j,n), and exhibited a much larger lateral displacement than the outer walls (Figure 1j; Figure S1l,m, Supporting Information). The jacket cell width significantly increased by 16.9% (Figure 1k), while the jacket cell length decreased slightly by 4.9% (Figure 1l). Thus, the cell width/length ratio increased substantially by 21.1% (Figure 1m). These results indicate that jacket cell geometry changed after antheridium burst in *P. patens*.

**Figure 2:**
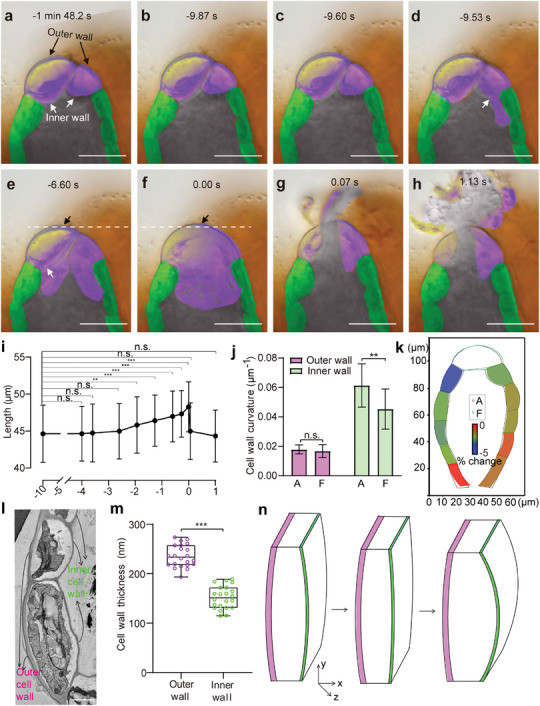
Dynamics of antheridium burst in *P. patens*. a–h) Antheridium burst processes by time lapse images. Antheridium burst is recorded at 15 frames per second: mature antheridium in water a–c); inner wall of one apical cell ruptured d), indicated by white arrow; another apical cell inner wall ruptured, as shown in white arrow e); apex swelling to maximum f); apex burst g) and sperms release h). Apex at maximum is defined as 0 s. Black arrows indicate outer cell walls of apex. The horizontal white dashed lines visualize the changes of boundary of outer cell walls of apex. The jacket cells and apical cell contents have been false‐shaded in green and purple, respectively, for clarity. The gray color indicates spermatogenous cells. i) Changes of outer wall length of apex during antheridium burst processes. Results are mean ± s.d. (*n* = 12); one‐way ANOVA with Tukey's test; **P < 0.01; ***P < 0.001; n.s., no significance (*P* > 0.05). j) Quantification of the curvature of the jacket cell walls before burst a) and apex at maximum f). Results are mean ± s.d. (*n* = 10); one‐way ANOVA with Dunnett's test; **P < 0.01, n.s., no significance (*P* > 0.05). k) Simulated changes of jacket cell shape before burst a) and apex at maximum f). Four antheridia are computed. Heat‐map indicates the reduction of jacket cell area. l) TEM images of jacket cells in the longitudinal section. m) The quantification of thickness of jacket cell walls. Box plots depict min to max values. Results are mean ± s.d. (*n* = 22); ***P < 0.001; Welch's *t*‐test. n) Illustration of how the jacket cell geometry triggers energy storage and release. Left panel: Inner walls bowed toward the cavity. Middle panel: the contents of apical cells swarming into the cavity led to the inner cell walls in compression and thereby enhanced the tension of the jacket cells. Right panel: the tension is released after burst. Outer wall (purple), inner wall (green). Scale bars, 20 µm in a–h), 2 µm in l).

To obtain more accurate changes of the entire cellular and organization geometry after antheridium burst in *P. patens*, we constructed 3D representations of the antheridium structure by combining a sequence of 1 µm thick sections in longitudinal direction (Figure 1o–r). Similar to 2D, the jacket cells had only small contractions in length, while largely expanding in width in both longitudinal and transverse directions (Figure 1s–w; Figure S1n, Supporting Information). Moreover, the inner walls had increased curvature after the burst (Figure 1x). We also detected slightly reduced antheridium volume but a large expansion in jacket cell volume, and the cavity volume tended to decrease substantially (Figure 1y; Figure S1o, Supporting Information). Thus, as antheridium dehiscence occurred the inner walls of jacket cells freely expanded toward the cavity (Figure 1g–i,j, and Figure 2h), which transformed the elastic potential energy into kinetic energy and caused the sperm cells to release. This process indicates that the changes in jacket cell geometry play an important role in the rapid release of sperm in *P. patens*.

### 2.2 Dynamics of Antheridium Burst in *P. Patens*

We identified the role of the inner cell walls of jacket cells in energy release, but the critical component for antheridium dehiscence of *P. patens* is the build‐up of energy. To address this mechanism, we tracked the process of antheridium burst in detail. Upon antheridium maturation of *P. patens*, the apical and jacket cells were highly vacuolated, which pushed the chloroplasts against the inner cell walls that enclose the spermatogenous cells (Figure 1c and **Figure** **2**a–c; Figure S2a,d, Supporting Information). After water application, the inner cell wall of one apical cell ruptured and the cellular contents of the apical cell were forced into the cavity (Figure 2d; Movie S4, Supporting Information). Subsequently, the inner wall of another apical cell burst, followed by the influx of cellular contents and water into the sperm chamber as well (Figure 2e). Almost at the same time, we observed a rapid swelling of the apex, and the length of its outer cell wall increased by ≈7.3% in 2.6 s (Figure 2f,i). After the rapid swelling, the outer wall of apex immediately ruptured and sperm cells were expelled (Figure 2g,h; Movie S4, Supporting Information).

**Figure 3:**
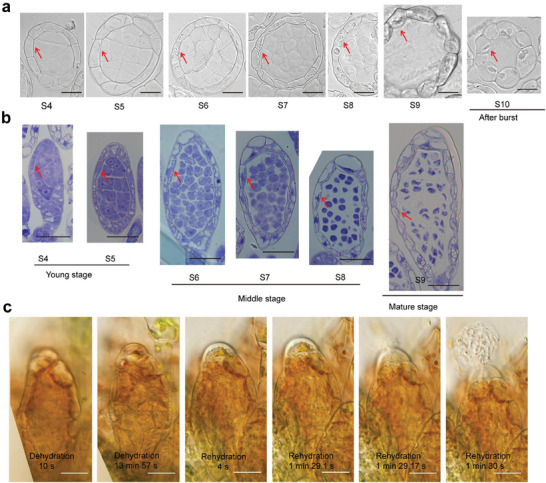
Hydrostatic pressure is required for sperm release in *P. patens*. a, b) The transverse section a) and longitudinal section b) from the antheridium at different developmental stages. Different stages were classified according to the previous study.^[^ ^8b^ ^]^ Red arrows indicate inner walls. c) Water is required for antheridium burst. Dehydration of mature antheridium using 30% sucrose and subsequent rehydration with pure water. Scale bars, 10 µm in a), 20 µm in b, c).

We next investigated the role of the jacket cell geometry in the build‐up of elastic energy. During antheridium maturation of *P. patens*, the inner walls of the jacket cells were not flat but rather bowed toward the spermatogenous cells with increased curvature and reduced angle between the adjacent jacket cell inner walls, suggesting increased turgor pressure in jacket cells (**Figure** **3**a,b; Figure S2a–d, Supporting Information). Upon application of water, the inner walls of the apical cells first burst in mature antheridium (Figure 2d,e). Water and cellular contents of apical cells swarmed into the cavity, which may increase the pressure in the sperm chamber (Figure 2d–f; Movie S4, Supporting Information). We also observed a reduction in the jacket cell area and the degree of inner wall curvature during this process (Figure 2f,j,k,n). In contrast, the curvature of outer walls did not change during this process (Figure 2f,j,k; Movie S4, Supporting Information). Thus, this process may promote tension in jacket cell walls to generate elastic energy. We further found that the jacket cell walls exhibited uneven thickness by transmission electron microscopy (TEM) analysis when the antheridium was mature, with the outer walls being considerably thicker than the inner walls (Figure 2l,m). In addition, jacket cells were stained with Calcofluor White that stains cellulose and callose,^[^ ^10^ ^]^ exhibiting higher fluorescence in the outer cell walls compared to the inner walls (Figure S3a, Supporting Information). Thus, these data suggest non‐uniform distribution of cell wall material properties in the jacket cells of *P. patens*. The thickness and rigid cellulose microfibrils can contribute to the reinforcement of plant cell walls. We hypothesized that the stiffer outer walls may prevent jacket cells from extending outwards, which may facilitate the build‐up of energy in confined space (Figure 2f,k). These results suggest that the stiffer outer walls of the jacket cells might provide a key mechanism that stores energy. Thus, once a sufficient amount of energy accumulated in the antheridium and the outer walls of apical cells burst, the pressure difference between the jacket cells and cavity is diminished thus relaxing the inner walls of the jacket cells (Figure 2n; Movie S4, Supporting Information).

### 2.3 Hydrostatic Pressure is Required for Sperm Release

The dehydration‐induced shrinkage of epidermal cells and the inextensible endothecium with lignified thickened walls are crucial to generate force for anther splitting in most seed plants.^[^ ^11^ ^]^ In this process, endothecium secondary thickening serves as an apparatus to store the energy to release pollen.^[^ ^12^ ^]^ However, sperm discharge in the majority of non‐seed plants requires the presence of water.^[^ ^5a–c^ ^]^ The hydrostatic pressure exerted in the sperm chamber could force the sperm mass out of the antheridium in bryophytes.^[^ ^7^, ^13^ ^]^ Thus, we further investigated whether the hydrostatic pressure is crucial for antheridium burst in *P. patens*. We monitored the changes of mature antheridia in 30% and 50% sucrose solutions for dehydration (Figure S2f,g, Supporting Information). Drying was characterized by clear plasmolysis in apical cells, antheridium shrinkage, and a reduction in cavity area in 30% sucrose solutions (Figure S2f,h, Supporting Information), indicating a potential reduction of pressure in apical and jacket cells as well as sperm chamber due to osmosis. Moreover, we found that the mature antheridium failed to burst under dehydration, but the burst occurred when it was subsequently rehydrated with pure water (Figure 3c), suggesting that sucrose may prevent the antheridium burst by alleviating the pressure in the jacket cells and cavity, leading to the failure of antheridium burst. Taken together, these findings demonstrate a water‐triggered mechanism for antheridium burst in moss, which is different from anther dehiscence that requires drying in most flowering plants.

### 2.4 Jacket Cell Geometry Changed in *ppvns4* Mutants

**Figure 4:**
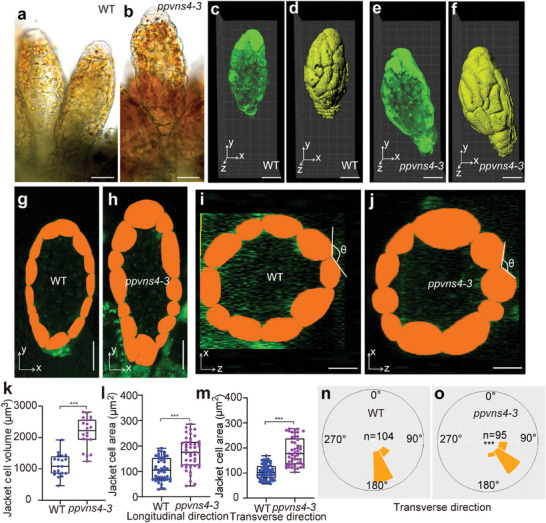
Jacket cell geometry altered in *P. patens ppvns4* mutant. a, b) Mature antheridium of the wild type a) and *ppvns4* mutant b). c–f) 3D fluorescence images of wild type c) and *ppvns4* mutant e), and 3D models of wild type d) and *ppvns4* mutant f). 3D models were reconstructed based on the fluorescence. g,h) Jacket cells in the X‐Y plane of mature antheridium in the wild type g) and *ppvns4* h). i,j) Jacket cells in the X‐Z plane at the middle region of mature antheridium in the wild type i) and *ppvns4* j). The jacket cells have been false‐shaded in orange for clarity (g–j). k–m) Quantitative analysis of jacket cell size. Jacket cell size parameters including volume k), area in the longitudinal direction l), and transverse direction m). Box plots depict min to max values. Results are mean ± s.d. (*n* ≥ 20); ***P < 0.001, Welch's *t*‐test. n, o) Quantification of angles shown in circular histograms in the wild type n) and *ppvns4* mutant o). The angle between outer walls of two jacket cells was shown in the wild type i) and *ppvns4* mutant j). Asterisks indicate significant differences compared to wild type, as determined by Welch's *t*‐test (***P < 0.001). Scale bars, 25 µm in a, b), 20 µm in c–j).

As the NAC family transcription factors, the NAC SECONDARY WALL THICKENING PROMOTING FACTORs (NSTs), the VASCULAR‐RELATED NAC‐DOMAIN (VND), and SOMBRERO (SMB) (VNS)‐related proteins, have been reported to regulate anther dehiscence and pod shattering through promoting secondary wall thickenings in Arabidopsis,^[^ ^12^, ^14^ ^]^ and the *P. patens* genome contains 8 *VNS* genes (Figure S4, Supporting Information).^[^ ^15^ ^]^ To identify the role of NAC transcription factors in antheridium dehiscence in *P. patens*, we searched a publicly available dataset^[^ ^16^ ^]^ and identified *PpVNS4* as significantly expressed in antheridia (Figure S5a, Supporting Information). This expression pattern was further confirmed in *PpVNS4‐GUS* lines (Figure S5b, Supporting Information). It has been shown that *ppvns4* mutants displayed enlarged central and transfer cells of the sporophyte foot.^[^ ^17^ ^]^ We reasoned that the geometry of antheridium in *ppvns4* mutants might be affected. As predicted, the shape of the antheridium and jacket cell in *ppvns4* mutant changed (**Figure** **4**a–j). The antheridium length and width were significantly larger in *ppvns4* mutants than wild type (Figure 4a–f; Figure S5e,f, Supporting Information). Altered organ size can be correlated to a change in cell size and/or cell number. In this regard, the jacket cell size parameters, including jacket cell volume and the area in the longitudinal and transverse directions, were notably higher in *ppvns4* compared to wild‐type jacket cells (Figure 4k–m), with a similar number of jacket cells in both directions (Figure S5h–j, Supporting Information). Moreover, a highly linear relationship between antheridium size and cell size was observed in the wild type and *ppvns4* mutant (Figure S5g, Supporting Information). These findings indicate that the changes in jacket cell size primarily contributed to changes in antheridium size caused by the *ppvns4*. We also observed that the outer walls of the jacket cells in *ppvns4* mutants expanded outward (Figure 4a,b,g–j), with the angles between the jacket cell outer walls being significantly larger in the wild type than the *ppvns4* mutant (Figure 4i,j,n,o). These differences indicate that the *PpVNS4* plays a role in the regulation of jacket cell geometry and antheridium size.

### 2.5 Cell Wall Mechanics Play a Role in Sperm Release

**Figure 5:**
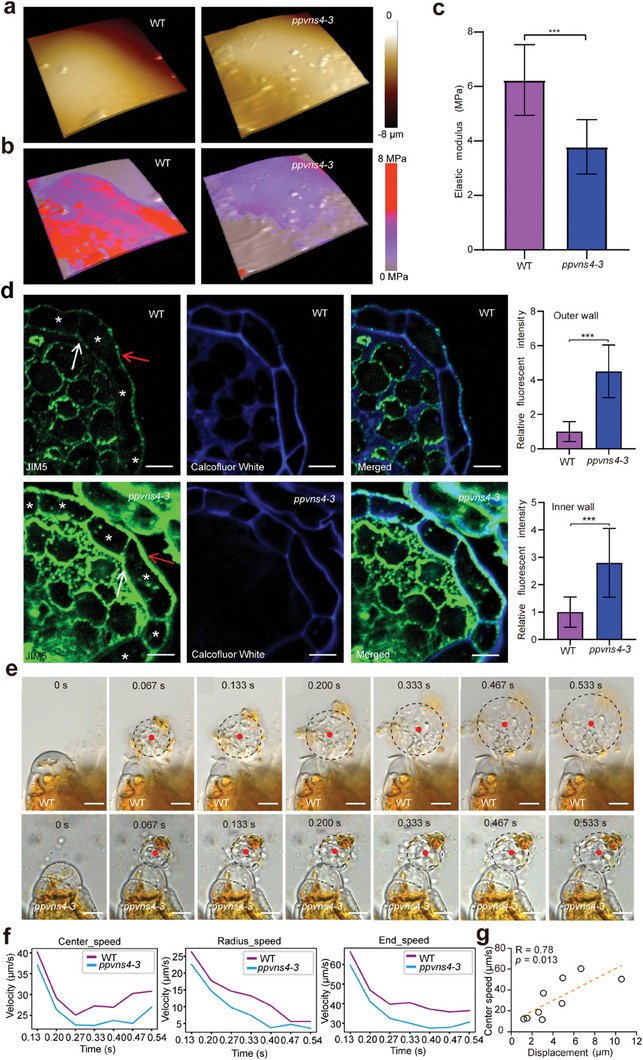
Jacket cell wall mechanics affect sperm release in *P. patens*. a) 3D topography of antheridium jacket cells of the wild type and *ppvns4* by AFM. Colors represent distance from base. b) Jacket cell topography overlaid with elastic modulus map of the wild type and *ppvns4*. Colors indicate elasticity. c) Quantification of elastic modulus on jacket cell walls of antheridium by AFM using the Peak Force QNM mode. Results are mean ± s.d. (n = 10); Welch's *t*‐test; ***P < 0.001. The average was measured in an 8‐µm × 8‐µm area of a cell with the highest modulus. d) Immunolabeling of antheridium with JIM5 for low‐methylesterified pectin epitope at S8 stage. Labeling of transverse sections of antheridium with JIM5 (green signal) and cellulose‐binding Calcofluor white (blue signal). Asterisks indicate jacket cells. Red arrow indicates the outer cell wall and white arrow indicates the inner cell wall. Quantification of relative fluorescent intensity detected by antibody JIM5. The mean value of fluorescence intensity of wild type is set as 1. Results are mean ± s.d. (*n* ≥ 22); Welch's *t*‐test; ***P < 0.001. e) The sperm release from antheridium in the wild type and *ppvns4*. The black dashed circles indicate sperm mass. Red dots indicate the center of the sperm mass. f) Quantification of speed of sperm mass release, as shown using the speed of center movement, radius expansion, and end sperm. *n* = 6. g) Correlation between center speed and displacement of outer walls after burst by linear regression analysis. The displacement of outer walls after antheridium burst was shown in Figure S1l (Supporting Information). Scale bars, 5 µm in d), 10 µm in e).

The VNS clade has been reported to positively regulate cell wall modifications in anthers and root cap cells in vascular plants.^[^ ^12^, ^18^ ^]^ Next, we explored the mechanics of the jacket cell walls in *P. patens*. We measured the elastic properties of the jacket cell wall using nanoindentation atomic force microscopy (AFM) to detect the surface structures and assess the stiffness.^[^ ^19^ ^]^ The elastic modulus of the jacket cell walls showed that the mechanical properties of outer walls were altered in the *ppvns4* mutants, and that the outer walls of the wild type exhibited an elastic modulus that was significantly higher than that of the *ppvns4* mutant (**Figure** **5**a–c). Furthermore, TEM analysis demonstrated no significant difference in cell wall thickness between the wild type and *ppvns4* mutant (Figure S7a,b, Supporting Information). These data suggested that the outer walls had reduced stiffness in the enlarged jacket cells of the *ppvns4* mutants. Together, these results suggest that the enlarged cells in *ppvns4* mutants might be associated with changed mechanical property of jacket cell walls.

Recently, pectin has been shown to play a critical role in regulating cell wall mechanics and cell size.^[^ ^19^, ^20^ ^]^ Homogalacturonan (HG) polysaccharides can represent up to 65% of pectin in primary walls, which are synthesized in the form of highly methylation and subsequently undergo demethylesterification by plant pectin methylesterases (PMEs).^[^ ^21^ ^]^ The increase in pectin demethylesterification could lead to the decrease of the stiffness of the cell walls.^[^ ^22^ ^]^ Furthermore, PME activity can be antagonized by endogenous PME inhibitors (PMEIs).^[^ ^23^ ^]^ For example, PMEI18 can regulate pectin demethylesterification and stomatal dynamics mainly through inhibiting PME31 in cell walls.^[^ ^24^ ^]^ Previous studies have uncovered that the expression of *PMEI3* and *PMEI18* is reduced in *nst1nst3* double knockout mutants, while it is significantly increased in Arabidopsis plants overexpressing *NST1* (Figure S5k, Supporting Information).^[^ ^12^, ^25^ ^]^ Coinciding with this, RNA‐seq analysis revealed that overexpression of *PpVNS4* resulted in up‐regulation of putative homologous genes to *PMEI* and selective down‐regulation of putative homologous genes to *PME* (Figure S6; Tables S2 and S3, Supporting Information). Thus, we focused on the distribution of pectin in different forms in the jacket cell walls of antheridium in *P. patens*. We performed immunolabeling using antibodies against low‐ or non‐esterified (JIM5) and highly methylesterified (JIM7) HG on transverse sections of the antheridium.^[^ ^26^ ^]^ The jacket cell walls of antheridium of *ppvns4* showed strong binding of JIM5, suggesting an increased abundance of unesterified pectin (Figure 5d). However, the distribution of the highly methylesterified pectin labeled by the JIM7 antibody in the jacket cell walls was comparable in the wild type and *ppvns4* mutant (Figure S7c,d, Supporting Information). Additionally, there is a selective up‐increase of putative homologous genes to *CesA*, a cellulose synthase subunit gene, in *PpVNS4* overexpression lines (Figure S6c, Supporting Information). We therefore investigated the changes in cellulose abundance in jacket cell walls of wild type and *ppvns4* mutant. The jacket cell walls stained with Calcofluor White in wild type exhibited a higher cellulose level compared to *ppvns4* mutant (Figure S3a,b, Supporting Information). Taken together, these data suggest that the abundance of low‐ or non‐esterified HG and the reduction of cellulose in the jacket cell walls may affect cell wall mechanics.

We further investigated whether the sperm release processes were affected in the *ppvns4* mutant by analyzing the ejected movement speed of the sperm mass and diffusion of sperm mass. We found that the ejection of the sperm mass was much slower in the *ppvns4* mutant than in the wild type (Figure 5e,f). Moreover, we observed a highly linear relationship between center speed and displacement of the outer wall (Figure 5g). These results indicated that the sperm release is impaired in *ppvns4* mutants. It is therefore tempting to speculate that the strengthened outer walls of jacket cells may be important to store hydrostatic energy for sperm release by maintaining a certain shape and size of the antheridium.

### 2.6 Sperm Release in Liverwort Marchantia Polymorpha (M. Polymorpha)

**Figure 6:**
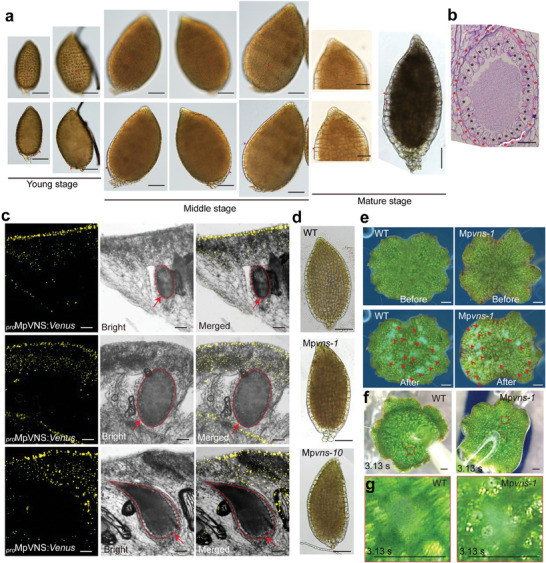
Sperm release is similar in *M. polymorpha* wild type and Mp*vns* mutants. a) Antheridia at different stages in *M. polymorpha*. Young stage: jacket cells are small and have dense cytoplasm. Middle stage: the antheridium is much larger than that in young stage, and the jacket cells are rectangular. Mature stage: the jacket cells are larger and becoming round. Arrows indicate jacket cells. b) The longitudinal section from mature antheridium in *M. polymorpha* with toluidine blue staining. Black asterisks indicate jacket cells. Red asterisks indicate surrounding cells. c) Expression analyses of the Mp*VNS* gene in *M. polymorpha* expressing the *Venus* gene driven by Mp*VNS* promoter (*_pro_* Mp*VNS:Venus*). From left to right, images of Venus fluorescence (yellow signal), bright‐field, and merged. The fluorescence was not detected in antheridia at the young stage (upper panel), middle stage (middle panel) and mature stage (lower panel), suggesting that *MpVNS* is not expressed in the antheridium. Red arrows and red dashed lines indicate antheridium. d) Mature antheridium is similar in wild type and Mp*vns* mutants. Isolated mature antheridium from antheridiophore in the wild type and Mp*vns* mutants. e) Sperm release is similar in the wild type and Mp*vns* mutant. Images of antheridiophore of WT and Mp*vns* before and after adding water to the antheridiophore for 2 min. Red arrowheads indicate the release of sperm cells (white cloud). f, g) Sperm release in the wild type and Mp*vns* mutants after adding water for 3.13 s. Red squares indicate released sperm mass in f). Magnified views g) of the regions enclosed in the red squares in f). Scale bars, 50 µm in a,b,c), 100 µm in d), 1 mm in e–g).

The antheridia are naked in most moss, liverworts and ferns,^[^ ^5^, ^6^, ^7^, ^13^, ^27^ ^]^ but the antheridia of *M. polymorpha* and its relatives are embedded inside of antheridial receptacles of the antheridiophore (Figure S8a, Supporting Information).^[^ ^27^, ^28^ ^]^ Unlike the shape of the jacket cells in *P. patens*, the jacket cells were round and swollen extensively in mature antheridia in *M. polymorpha* (**Figure** **6**a,b). The size of the isolated mature antheridium in *M. polymorpha* increased after it was exposed to water (Figure S8b, Supporting Information). When we applied water to the antheridiophore of *M. polymorpha*, sperm cells were immediately released from the antheridia (Figure 6e–g). However, sperms failed to discharge from an isolated mature antheridium of *M. polymorpha* in pure water (Movie S5, Supporting Information). These findings support the notion that like the jacket cell outer walls of *P. patens*, the surrounding cells of the antheridium in *M. polymorpha* (Figure 6b; Figure S8a, Supporting Information) might play an equivalent role in the storage of energy by preventing jacket cells from expanding when hydration, thereby leading to sperm release.

Given that the *M. polymorpha* genome contains a single VNS gene (Figure S4, Supporting Information),^[^ ^15^ ^]^ we next investigated whether the *MpVNS* gene plays a role in sperm release in *M. polymorpha*. We found that *MpVNS* is not expressed in the antheridium of *M. polymorpha* by searching publicly available data (Figure S8c,d, Supporting Information),^[^ ^29^ ^]^ which was confirmed by analyzing *M. polymorpha* plants stably expressing the *Venus* gene under control of *MpVNS* promoter (Figure 6c). Coinciding with these results, antheridia development and sperm release were not affected in Mp*vns* mutants (Figure 6d–g). Our results suggested that the *VNS* gene did not play a role in sperm release in *M. polymorpha*, which might be associated with antheridia embedded in the receptacle.

## 3 Conclusion

During terrestrialization process, the plants undergo a set of morphological changes to better adapt to diverse environmental conditions such as drought.^[^ ^30^ ^]^ Mosses and other bryophytes are believed to represent early divergent land plants with haploid reproductive organs,^[^ ^31^ ^]^ which is separated from the flowing plants by 450 million years.^[^ ^32^ ^]^ The bryophytes and flowing plants have evolved different reproductive strategies to ensure successful fertilization, and the bryophytes possess many unique traits that do not exist in most flowering plants.^[^ ^28^ ^]^ In the 1910, antheridium dehiscence to immediately release the sperms was described in *Sphagnum cymbifolium*.^[^ ^33^ ^]^ Since then, antheridium dehiscence under water was found to discharge the spermatocytes in a mass in the majority of the bryophytes, including *Minum hornum*, *Bryum argenteum*, *Funaria hygrometrica*, and *Bryum capillare*.^[^ ^7^, ^9^ ^]^ However, the underlying biological and mechanical mechanisms of antheridium burst are still unknown. Here, our results demonstrated that, as a morpho‐mechanical innovation, a single layer of jacket cells of the antheridium in *P. patens* could efficiently store hydrostatic energy in a confined space on hydration and then release sperm cells. Our results revealed that turgor pressure could be important for tension generation in jacket cell walls. Moreover, in the presence of water, the burst of the apex inner walls leads to water and apical cellular contents into the sperm cavity, potentially enhancing the tension in the jacket cell walls. Subsequently, the release of pressure and the expansion of the inner wall toward the cavity lead to the ejection of sperm cells. Hence, the geometry of the jacket cells provides a mechanism by which elastic energy is both stored and released, in which a strengthened jacket cell outer walls involving the PpVNS4 protein may play a crucial role. In contrast, drying is required for anther dehiscence in most seed plants to release pollens.^[^ ^11b^ ^]^ The biomechanics of mature anther dehiscence in most seed plants depend on the bilayer system, which consists of the outer epidermal cell layer and the secondary cell wall thickening of endothecium that serves as the mechanical layer. Dehydration reduces the turgor pressure of the epidermal cells, leading to the decrease of natural length of the epidermis. However, the secondary thickening prevents shrinkage of endothecium, generating mechanical force to cause it to bend outwards for pollen release in dry environments.^[^ ^11^ ^]^ Anther dehiscence was impaired in Arabidopsis mutants *nst1nst2* and *myb26* (At3g13890) that have reduced secondary thickening in the endothecium.^[^ ^12^, ^34^ ^]^ However, in *M. polymorpha* the VNS protein is not involved in sperm release. Thus, these data suggest that the *VNS* gene may be co‐opted in the evolution of morphological traits to direct male gametes discharge at optimal conditions in moss and seed plants. Additionally, unlike the antheridium that is generally naked in moss *P. patens*, the antheridia of liverwort *M. polymorpha* and its relatives are produced singly to be embedded within the antheridial chamber that is sunk in the tissue of receptacle.^[^ ^28^, ^29^, ^35^ ^]^ By contrast, the antheridia are naked in other liverworts, such as *Fossombroniaceae*.^[^ ^27c,d,g^ ^]^ It is therefore tempting to speculate that the antheridium of *M. polymorpha* may adopt a different strategy for sperm release. Like the jacket cell outer walls of *P. patens*, the cells surrounding the antheridium in *M. polymorpha* may play a similar role in the storage of energy. Thus, our results provide insights into the morphological changes in diverse reproductive systems for dehiscence and the adaptive evolution of plants.

## 4 Experimental Section

### Plant Materials and Growth Conditions

The Gransden (Gd) strain of *P. patens* was used as wild type for this study.^[^ ^32^ ^]^ The mutants were generated in Gd background. For protonemata growth, *P. patens* were cultured on BCDAT media with 0.8% (w/v) agar at 25 °C under continuous white light. For gametophore growth, protonemata were transplanted to autoclaved peat pellets (Jiffy‐7, Jiffy Products International AS, Kristansand, Norway) at 25 °C under continuous white light. For antheridia development, the gametophores were grown at 15 °C with 8:16 h light/dark photoperiod.^[^ ^16^, ^36^ ^]^ *PpVNS4‐GUS* lines were obtained from previous published report.^[^ ^17^ ^]^ The “Cambridge” (Cam) strain of *M. polymorpha* was used as wild type and the mutants were generated in Cam background.^[^ ^37^ ^]^ The gemmae and thalli were cultured on half‐strength Gamborg's B5 media containing 1% (w/v) agar at 22 °C under long‐day conditions (16:8 h light/dark conditions). To induce reproductive organs, plants with 3–4 weeks of age were transferred to soil under far red light (Philips).

### Plasmid Construction and Plant Transformation

To construct the *ppvns* mutants, the genomic DNA of *P. patens* was used as a template. Genomic fragments of ≈1000 bp upstream and 1000 bp downstream of *PpVNS4* (*Pp3c6_1310V3.1*) coding regions were amplified via PCR and inserted into the pTN182 plasmid. For generation of estrogen‐inducible *PpVNS4* overexpression lines, gateway *rfcA* fragment of pPGX8 vector (AB537482) was replaced by *Apa*I restriction sites.^[^ ^38^ ^]^ The coding region of *PpVNS4* was then cloned into this altered pPGX8 vector. PEG‐mediated protoplast transformation was performed to generate transgenic *P. patens*.^[^ ^39^ ^]^ PCR was performed to identify transgenic plants with the targeted DNA fragment (Figure S5c, Supporting Information). To detect if there were unexpected substitutions or insertions in these transgenic lines, the relative copy number of recombinant DNA fragments was quantified (Figure S5d, Supporting Information).^[^ ^40^ ^]^ *PpEF1α* (*Pp3C2_10310V3.1*) was used as a reference gene. *MpVNS* (*Mp6g20920*) is the sole VNS gene in *M. polymorpha* from phylogenetic analysis. To construct Venus fused with nuclear localization signal peptide lines (NLS) driven by the *MpVNS* native promoter, the flanking 4 kb sequence upstream of the start codon of *MpVNS* (*proMpVNS*) was amplified by PCR from the *M. polymorpha* genome, and then cloned into pGreen plasmid.^[^ ^41^ ^]^ To generate a single Mp*vns* mutant (Figure S9, Supporting Information), we used the CRISPR/Cas9 system. One oligonucleotide pair was annealed and cloned into the MH‐MCS‐U3 vector,^[^ ^42^ ^]^ in which the U3 promoter was replaced by the *M. polymorpha U6* promoter. This construct was transformed into Cam wild‐type sporelings as previously reported.^[^ ^43^ ^]^ The primers used were listed in Table S1 (Supporting Information).

### Microscopy Analysis

Antheridia were manually isolated from gametophore apexes as previously reported.^[^ ^44^ ^]^ The isolated antheridia with stem were immobilized vertically on a 0.8% (w/v) agar plate overnight. For visualization of antheridium burst and sperm release, the antheridia were placed on a microscope slide with pure water and the images were immediately acquired on a microscope (Olympus BX53), equipped with an Olympus DP80 camera. For Cryo‐SEM, samples were frozen in subcooled liquid nitrogen (−210 °C) and transferred into a vacuum cabin to the cold stage, and underwent sputter coating with platinum. Images were then acquired with a Regulus 8100 (Hitachi Co., Ltd., Japan). For obtaining the volume of samples, the images of the 3D Z‐stacks were acquired using the two‐photon Olympus FV1000MPE microscope. Image acquisition was performed with excitation at 720 nm and emission at 420–460 nm. Stacks were collected at a 1 µm slice interval in longitudinal direction, stepping through the entire antheridium. Z‐stacks were processed using IMARIS9.5.0 software to render the 3D models to visualize the data.

Antheridia were fixed with 4% (w/v) formaldehyde freshly prepared from paraformaldehyde in 100 mm sodium phosphate buffer (pH 7.0) overnight at 4 °C. The samples were dehydrated using a graded ethanol series (30, 50, 70, 80, 90, 100, 100% (v/v)) for 20 min at each step. The dehydrated antheridia were then infiltrated with progressively higher concentrations of LR White resin diluted with ethanol (30, 50, 100, 100, 100% (v/v) resin) for 24 h each. The infiltrated antheridia were embedded in LR‐White resin (London Resin Co.) and polymerized at 60 °C for 24 h. Semi‐thin transverse Sections (2 µm) were obtained using a Leica Ultracut R. The sections were stained with 0.1% (w/v) Toluidine Blue O at 80 °C for 5 s, and the images were acquired using an Olympus BX53‐DIC microscope. For immunolabeling, semi‐thin transverse Sections (2 µm) were blocked with 3% (w/v) bovine serum albumin (BSA) in phosphate‐buffered saline (PBS) solution and then incubated with the corresponding antibodies. The rat JIM5 and JIM7 were used at 1:40 dilution.^[^ ^45^ ^]^ Secondary antibody (goat anti‐rat‐FITC) was diluted 1:1000 in the blocking solution, and sections were incubated with the secondary antibody in darkness for 2 h at room temperature. The sections were washed in PBS 10 times and then incubated with Calcofluor White (0.02% (w/v). The Calcofluor White can stain cellulose and callose,^[^ ^10^ ^]^ allowing for detection of cell walls. FITC and Calcofluor White were imaged using confocal laser microscope (Zeiss LSM800) at 488 and 405 nm for excitation, respectively, and 498–551 nm (FITC) and 427–471 nm (Calcofluor White) for detection, respectively. The images of wild type and *ppvns4* mutant were acquired at the same microscope settings with same frame time and same image processing settings. For Calcofluor White staining, the images of wild type and *ppvns4* mutants were collected over a 134.69 by 134.69 µm image size with a resolution of 1024 × 1024 pixels. The frame time was 10.07 s. The pinhole was 1.00 AU/51 µm. Detector grain and detector offset were 639 V and 0, respectively. For JIM5 immunolabeling and JIM7 immunolabeling, the images of wild type and *ppvns4* mutants were collected over a 134.69 by 134.69 µm image size with a resolution of 2048 × 2048 pixels. The frame time was 40.27 s. The pinhole was 1.00 AU/59 µm. Detector grain and detector offset were 577 V and 0, respectively. The fluorescence intensity was measured as shown in Figure S3c (Supporting Information).

For TEM analysis, antheridia were fixed with 2% (v/v) glutaraldehyde and 2% (w/v) formaldehyde freshly prepared from paraformaldehyde in a 50 mm sodium cacodylate buffer (pH 7.4) at 4 °C overnight. The samples were then post‐fixed with 1% (v/v) osmium tetroxide (OsO_4_) and 1.5% potassium ferricyanide in a 50 mm cacodylate buffer (pH 7.4) overnight. The samples were then treated with 1% thiocarbohydrazide for 30 min at room temperature, followed by 1% OsO_4_ for 1 h at room temperature. After incubation in 50 mm maleate buffer (pH 5.2) with 2% uranyl acetate, the samples were dehydrated in an ethanol series. The dehydrated samples were embedded in Quetol 651 resin (Agar Scientific). The 70 nm thin sections were cut with a Leica Ultracut R and examined using a JEM‐1230. For cell wall thickness, regions where the outer wall edge was parallel to the inner wall edge were selected for measurement using the ImageJ software (http://rsb.info.nih.gov/ij/).^[^ ^46^ ^]^

Venus fluorescence of proMp*VNS*:Venus‐NLS was observed by microscope. The antheridiophores were fixed in 4% (w/v) paraformaldehyde in 100 mm sodium phosphate buffer (pH7.0) and then embedded in 5% (w/v) agar in PBS. The 100 µm sections were obtained with a vibratome (Leica VT1000S). Fluorescence and bright‐field images were acquired under an Olympus BX53 microscope.

### AFM Measurements

The antheridia were treated with 30% sucrose for 10 min to remove turgor pressure and then attached to a glass slide using transparent nail polish. The antheridium was scanned using a BioScope Resolve atomic force microscope (Bruker, Billerica, MA, USA). The probe was a standard pyramidal silicon nitride ScanAsyst‐Fluid cantilever (Bruker) with 0.7 N m^−1^ spring constant and 20 nm tip radius. The “PeakForce Quantitative Nanoscale Mechanical (QNM) in fluid” operation mode was used to record peak force error and the DMT modulus. Peak force frequency was set at 1 kHz and peak force set‐point at 3 nN. The images were acquired at 15 by 15 µm. The scanning rate was 0.5 Hz. The cell wall Young's modulus was measured using an indentation depth of 150 nm. Data were analyzed with NanoScope Analysis version 1.8. For statistical analysis of AFM, the position with the highest modulus in the outer walls was initially selected, and subsequently an 8‐µm × 8‐µm area surrounding this highest modulus position was selected. The average elastic modulus within this selected 8‐µm × 8‐µm area was then measured.

### Dynamics of Antheridium After Burst

To describe the dynamics of the antheridium after burst, the geometry of the antheridium jacket cells was computed during antheridium burst. Three distinct configurations of the jacket cell were considered. The jacket cell inner wall of antheridium is curved (convex) toward the cavity at the mature stage. Once the inner wall of the apical cell ruptured and the cell contents swarmed into the cavity, the inner walls must flatten. Once the antheridium burst, the inner wall was largely convex. The changes in width were much larger than the changes in length after the burst. The discretized points along the jacket cell wall were tracked using time‐lapse imaging analysis. The geometry was computed as:

Denoting the point i of the antheridium k at the time t by its coordinate $\left(x_{i}^{k}\left(t\right),y_{i}^{k}\left(t\right)\right)$, the antheridium geometry at the time *t* is represented by:

(1) $$geometry^{k}\;\left(t\right)=\left\{\left(x_{1}^{k}\left(t\right),y_{1}^{k}\left(t\right)\right),\text{…},\left(x_{i}^{k}\left(t\right),y_{i}^{k}\left(t\right)\right),\text{…}\left(x_{n}^{k}\left(t\right),y_{n}^{k}\left(t\right)\right)\right\}\;$$

Due to the various antheridium orientations recorded, the antheridium geometry is normalized to an upward perpendicular direction as shown in Figure S2e (Supporting Information). To do so, the point on the bottom left of antheridium was denoted as $\left(x_{l}^{k}\left(t\;=\;1\right),y_{l}^{k}\left(t\;=\;1\right)\right)$ and the point on the bottom right was marked as $\left(x_{r}^{k}\left(t\;=\;1\right),y_{r}^{k}\left(t\;=\;1\right)\right)$. The two points form a line that intersects with the x‐axis, forming an angle denoted by θ^*k*^(*t*) and the center between the two bottom points denoted by *c^k^* (*t*) are calculated as follows:

(2) $$\theta^{k}\left(t\right)\;=\;arctan\left(\frac{y_{r}^{k}\left(t\;=\;1\right)-y_{l}^{k}\left(t\;=\;1\right)}{x_{r}^{k}\left(t\;=\;1\right)-x_{l}^{k}\left(t\;=\;1\right)}\right)\in \left[-\frac{\pi }{2},\frac{\pi }{2}\right]$$

(3) $$c^{k}\left(t\right)\;=\left(\frac{x_{r}^{k}\left(t=1\right)+x_{l}^{k}\left(t=1\right)}{2},\frac{y_{r}^{k}\left(t=1\right)+y_{l}^{k}\left(t=1\right)}{2}\right)\;$$

The center is further set as the origin (0, 0) in the coordinate, and the antheridium geometry is clockwise rotated by θ^*k*^(*t*). Thus, the normalized geometry is obtained according to Equation (3), where the new geometries of both bottoms are symmetrically aligned on the x‐axis and the apical cell is on the top.

(4) $$geometry_{normal}^{k}\;\left(t\right)=\left[\begin{array}{cc} cos\;\theta^{k}\left(t\right) & sin\;\theta^{k}\left(t\right)\\ -sin\;\theta^{k}\left(t\right) & cos\;\theta^{k}\left(t\right) \end{array}\right]\;\left(geometry^{k}\left(t\right)-c^{k}\left(t\right)\right)\;$$

Taking the averaged shape of all the antheridium as the geometry model, denoted by *geometry*_model_(*t*), the dynamics of the antheridium after burst can be obtained using Equation (5), where *t*  =  0, 1 indicate the moments right before and after the burst.

(5) $$\mathrm{dynamics}\;=\;geometry_{model}\left(t\;=\;1\right)-\;geometry_{model}\left(t\;=\;0\right)$$

### Ejected sperm velocity

Here, we used circles to represent the ejected sperm mass, and the sperm mass’ movement and the expending speeds were used to compare the sperm ejecting velocities of the *P. patens* and *ppvns4* mutant (6 antheridia for both types are used for modeling). For each antheridium *k* at time *t*, the sperm mass was manually outlined by a circle, and the center of sperm mass was defined as (*x^k^* (*t*),*y^k^* (*t*)), with the radius of the sperm mass being *r^k^* (*t*). The movement of an ejected sperm mass is very complex, as it consists of movement of the sperm mass and the spread of individual sperms. So, we used three indexes to describe sperm mass movement: Center_speed represents an average speed of the center of sperm mass; Radius_speed represents an average speed of sperm mass spread; End_speed represents an additive effect of Center_speed and Radius_speed, which indicates the speed of sperm mass first ejected at the initial stage.

(6) $$Center\_speed^{*}\left(t\right)\;=\left(x^{k}\left(t\right)-x^{k}\left(t-1\right),y^{k}\left(t\right)-y^{k}\left(t-1\right)\;\right)/time\_step\;$$

(7) $$Radius\_speed^{*}\left(t\right)\;=\left(r^{k}\left(t\right)-r^{k}\left(t-1\right)\;\right)/time\_step\;$$

(8) $$\mathrm{End}\_\mathrm{spee}d^{*}\left(t\right)\;=\mathrm{Cent}\mathrm{er}\_\mathrm{spee}d^{*}\left(t\right)+\mathrm{Radi}\mathrm{us}\_\mathrm{spee}d^{*}\left(t\right)\;$$

To minimize the error caused by the timestamp misalignment, we adopted the mean filter to smooth the speed curve where the speed at each time step is averaged by the adjacent two steps.^[^ ^47^ ^]^

(9) $$\begin{array}{ccc} Center\_speed\left(t\right)\; & = & \left(Center\_speed^{*}\left(t-1\right)+Center\_speed^{*}\left(t\right)\right.\\ & & \left.+Center\_speed^{*}\left(t+1\right)\right)/3\; \end{array}$$

(10) $$\begin{array}{ccc} Radius\_speed\left(t\right)\; & = & \;\left(Radius\_speed^{*}\left(t-1\right)+Radius\_speed^{*}\left(t\right)\right.\\ & & \left.+Radius\_speed^{*}\left(t+1\right)\right)/3 \end{array}$$

(11) $$\begin{array}{ccc} End\_speed\left(t\right)\; & = & \left(End\_speed^{*}\left(t-1\right)+End\_speed^{*}\left(t\right)\right.\\ & & \left.+End\_speed^{*}\left(t+1\right)\right)/3\; \end{array}$$

### Individual Sperm Release Velocity

The position of the sperm cells close to these cellular contents was specified as the initial point. The initially released speed of sperms was then calculated.

### Gene Expression Analysis

To analyze the expression of genes in *PpVNS4* overexpression lines, the 4‐day‐old protonemata of estrogen‐inducible *PpVNS4* overexpressor were treated with or without 1 µm β‐estradiol solution for 18 h. For each treatment, three biological replicates were tested. Total RNAs were extracted using the RNAprep Pure plant kit (Qiagen) following manufacturer's instructions. RNA‐seq libraries were constructed and then sequenced on Illumina NovaSeq6000. To analyze the expression of *PpVNS4* in different tissues of *P. patens*, we retrieved the RNA‐seq reads from published data.^[^ ^16^ ^]^ To analyze the expression of *MpVNS* in different *M. polymorpha* tissues, we retrieved the RNA‐seq reads from published data.^[^ ^29^ ^]^ *P. patens* genome annotation v3.3 were retrieved from Phytozome (https://phytozome‐next.jgi.doe.gov/info/Ppatens_v3_3). *M. polymorpha* genome annotation was retrieved from Phytozome (https://phytozome‐next.jgi.doe.gov/info/Mpolymorpha_v3_1). The reads were then mapped to the corresponding reference genome using HISAT2 (V2.2.1) with default parameters.^[^ ^48^ ^]^ The mapped reads were then sorted, indexed, and compressed using SAMtools.^[^ ^49^ ^]^ PCR duplicates were removed using Picard (v1.72) (http://broadinstitute.github.io/picard). The edgeR and StringTie was used to estimate gene expression levels.^[^ ^50^ ^]^

### Phylogenetic Analysis

Arabidopsis VNS protein sequences were used as queries to perform BLASTP analysis (E‐value threshold:1e‐5) and the hmmsearch in HMMER v3.3114 (–domE 0.001) against peptides annotated in the selected plants: *Anthoceros angustus*, *M. polymorpha*, *P. patens*, *Sphagnum fallax*, *Selaginella moellendorffii*, *Amborella trichopoda*, *Populus trichocarpa*, *Arabidopsis thaliana*, *Oryza sativa*, *Glycine max*, *Zea mays*, *Pinus taeda*. 97 sequences were retrieved and then aligned using MAFFT L‐INS‐i v7.490.^[^ ^51^ ^]^ Positions with gaps of ≥ 10% were removed using trimAl.^[^ ^52^ ^]^ The phylogenetic trees were constructed using the maximum likelihood (ML) method by RAxML (v8.2.12) using the WAG model.^[^ ^53^ ^]^ The reliability of the tree was tested with 1000 bootstrap replicates. The Interactive Tree Of Life (iTOL) (https://itol.embl.de) was used for phylogenetic tree display.^[^ ^54^ ^]^ The MEME v5.5.2 (https://meme‐suite.org/meme/tools/meme) was applied to find conservative motifs among VNS family members. The maximum number of motifs was set as 10 and the other parameters were default. TBtools was used to draw the motifs.^[^ ^55^ ^]^

## Conflict of Interest

The authors declare no conflict of interest.

## Author Contributions

X.Z., A.B., J.Y. and Y.L. contributed equally to this work. X.Z. and Q.Z. conceived the work and designed the experiments. X.Z. performed most of the experiments with contributions from Y.L., Z.Z. and M.Y.. A.B. conducted analysis of the antheridium dynamics. J.Y. performed the RNA‐seq analysis. Y.L. performed the acquisition of fluorescence images. S.Y. reconstructed the phylogenetic tree. X.Z., A.B., J.Y., Y.L. and M.Y. analyzed data. X.Z. and Q.Z. wrote the manuscript. All authors edited the manuscript.

## Supporting information

Supporting Information

Supplemental Movie 1

Supplemental Movie 2

Supplemental Movie 3

Supplemental Movie 4

Supplemental Movie 5

Supplemental Table 1

## Data Availability

The data that support the findings of this study are available from the corresponding author upon reasonable request.

## References

[1] a) H. Hofhuis , D. Moulton , T. Lessinnes , A.‐L. Routier‐Kierzkowska , R. J. Bomphrey , G. Mosca , H. Reinhardt , P. Sarchet , X. Gan , M. Tsiantis , Y. Ventikos , S. Walker , A. Goriely , R. Smith , A. Hay , Cell 2016, 166, 222; 27264605 10.1016/j.cell.2016.05.002 PMC4930488

[2] a) Y. Forterre , J. M. Skotheim , J. Dumais , L. Mahadevan , Nature 2005, 433, 421; 15674293 10.1038/nature03185

[3] J. M. Skotheim , L. Mahadevan , Science 2005, 308, 1308. 15919993 10.1126/science.1107976

[4] A. Galstyan , A. Hay , Curr. Opin. Genet. Dev. 2018, 51, 31. 29753214 10.1016/j.gde.2018.04.007

[5] a) A. F. Dyer , J. G. Duckett , The experimental biology of bryophytes, Academic Press, London 1984;

[6] a) D. J. Paolillo , Bioscience 1981, 31, 367;

[7] a) M. Hiss , R. Meyberg , J. Westermann , F. B. Haas , L. Schneider , M. Schallenberg‐Rüdinger , K. K. Ullrich , S. A. Rensing , Plant J. 2017, 90, 606; 28161906 10.1111/tpj.13501

[8] a) R. Kofuji , Y. Yagita , T. Murata , M. Hasebe , Philoso. Transac. Royal Soc. B: Bio. Sci. 2018, 373, 20160494; 10.1098/rstb.2016.0494 PMC5745330 29254959

[9] N. Cronberg , R. Natcheva , H. Berggren , Bryology in the New Millennium 2008, 347.

[10] a) R. Wang , H. A. Owen , A. A. Dobritsa , Plant Physiol. 2021, 187, 2393; 34890458 10.1093/plphys/kiab426 PMC8644823

[11] a) M. R. Nelson , L. R. Band , R. J. Dyson , T. Lessinnes , D. M. Wells , C. Yang , N. M. Everitt , O. E. Jensen , Z. A. Wilson , New Phytologist 2012, 196, 1030; 22998410 10.1111/j.1469-8137.2012.04329.x PMC3569878

[12] a) N. Mitsuda , M. Seki , K. Shinozaki , M. Ohme‐Takagi , The Plant Cell 2005, 17, 2993; 16214898 10.1105/tpc.105.036004 PMC1276025

[13] D. J. Paolillo , Am. J. Bot. 1977, 64, 81.

[14] N. Mitsuda , M. Ohme‐Takagi , Plant J. 2008, 56, 768. 18657234 10.1111/j.1365-313X.2008.03633.x

[15] Y. Nakano , M. Yamaguchi , H. Endo , N. A. Rejab , M. Ohtani , Front. Plant Sci. 2015, 6, 288. 25999964 10.3389/fpls.2015.00288 PMC4419676

[16] a) V. Sanchez‐Vera , K. Landberg , M. Lopez‐Obando , M. Thelander , U. Lagercrantz , R. Muñoz‐Viana , A. Schmidt , U. Grossniklaus , E. Sundberg , New Phytol. 2022, 233, 2614; 34942024 10.1111/nph.17938

[17] B. Xu , M. Ohtani , M. Yamaguchi , K. Toyooka , M. Wakazaki , M. Sato , M. Kubo , Y. Nakano , R. Sano , Y. Hiwatashi , T. Murata , T. Kurata , A. Yoneda , K. Kato , M. Hasebe , T. Demura , Science 2014, 343, 1505. 24652936 10.1126/science.1248417

[18] T. Bennett , A. van den Toorn , G. F. Sanchez‐Perez , A. Campilho , V. Willemsen , B. Snel , B. Scheres , The Plant Cell 2010, 22, 640. 20197506 10.1105/tpc.109.072272 PMC2861445

[19] a) S. A. Braybrook , E. K. Paluch , Biophysical Methods in Cell Biology (Ed.: E. K. Paluch ) Academic Press, Cambridge 2015;

[20] a) H. Zhang , Z. Guo , Y. Zhuang , Y. Suo , J. Du , Z. Gao , J. Pan , L. Li , T. Wang , L. Xiao , G. Qin , Y. Jiao , H. Cai , L. Li , Plant Cell 2021, 33, 581; 33955485 10.1093/plcell/koaa049 PMC8136896

[21] D. Mohnen , Curr. Opin. Plant Biol. 2008, 11, 266. 18486536 10.1016/j.pbi.2008.03.006

[22] A. Peaucelle , R. Wightman , H. Höfte , Curr. Biol. 2015, 25, 1746. 26073136 10.1016/j.cub.2015.05.022

[23] J. Pelloux , C. Rustérucci , E. J. Mellerowicz , Trends Plant Sci. 2007, 12, 267. 17499007 10.1016/j.tplants.2007.04.001

[24] X. Zhang , H. Guo , C. Xiao , Z. Yan , N. Ning , G. Chen , J. Zhang , H. Hu , Plant Physiol. 2023, 1, 603. 10.1093/plphys/kiad145 PMC10231589 36879425

[25] N. Mitsuda , A. Iwase , H. Yamamoto , M. Yoshida , M. Seki , K. Shinozaki , M. Ohme‐Takagi , Plant Cell 2007, 19, 270. 17237351 10.1105/tpc.106.047043 PMC1820955

[26] J. P. Knox , P. J. Linstead , J. King , C. Cooper , K. Roberts , Planta 1990, 181, 512. 24196931 10.1007/BF00193004

[27] a) D. J. Paolillo , New Phytologist 1975, 74, 287;

[28] B. Goffinet , W. R. Buck , A. J. Shaw , Bryophyte Biology 2009, 2, 55.

[29] a) A. Higo , M. Niwa , K. T. Yamato , L. Yamada , H. Sawada , T. Sakamoto , T. Kurata , M. Shirakawa , M. Endo , S. Shigenobu , Plant Cell Physiol. 2016, 57, 325; 26858289 10.1093/pcp/pcw005

[30] a) S. A. Rensing , Curr. Opin. Plant Biol. 2018, 42, 49,; 29525128 10.1016/j.pbi.2018.02.006

[31] C. H. Wellman , P. L. Osterloff , U. Mohiuddin , Nature 2003, 425, 282. 13679913 10.1038/nature01884

[32] S. A. Rensing , D. Lang , A. D. Zimmer , A. Terry , A. Salamov , H. Shapiro , T. Nishiyama , P.‐F. Perroud , E. A. Lindquist , Y. Kamisugi , T. Tanahashi , K. Sakakibara , T. Fujita , K. Oishi , T. Shin‐I , Y. Kuroki , A. Toyoda , Y. Suzuki , S.‐i. Hashimoto , K. Yamaguchi , S. Sugano , Y. Kohara , A. Fujiyama , A. Anterola , S. Aoki , N. Ashton , W. B. Barbazuk , E. Barker , J. L. Bennetzen , R. Blankenship , et al., Science 2008, 319, 64. 18079367 10.1126/science.1150646

[33] F. Cavers , Knowledge 1910, 7, 294.

[34] C. Yang , J. Song , A. C. Ferguson , D. Klisch , K. Simpson , R. Mo , B. Taylor , N. Mitsuda , Z. A. Wilson , Plant Physiol. 2017, 175, 333. 28724622 10.1104/pp.17.00719 PMC5580765

[35] M. Shimamura , Plant Cell Physiol. 2016, 57, 230. 26657892 10.1093/pcp/pcv192

[36] a) K. Sakakibara , T. Nishiyama , H. Deguchi , M. Hasebe , Evol. and Develop. 2008, 10, 555; 10.1111/j.1525-142X.2008.00271.x 18803774

[37] B. Pollak , M. Delmans , J. Haseloff , J. L. Bowman , T. Kohchi , K. T. Yamato , J. Jenkins , S. Shu , K. Ishizaki , S. Yamaoka , R. Nishihama , Y. Nakamura , F. Berger , C. Adam , et al., Cell 2017, 171, 287.28985561

[38] M. Kubo , A. Imai , T. Nishiyama , M. Ishikawa , Y. Sato , T. Kurata , Y. Hiwatashi , R. Reski , M. Hasebe , PLoS One 2013, 8, e77356. 24086772 10.1371/journal.pone.0077356 PMC3785464

[39] K. Sakakibara , T. Nishiyama , N. Sumikawa , R. Kofuji , T. Murata , M. Hasebe , Development 2003, 130, 4835. 12917289 10.1242/dev.00644

[40] B. Khraiwesh , M. A. Arif , G. I. Seumel , S. Ossowski , D. Weigel , R. Reski , W. Frank , Cell 2010, 140, 111. 20085706 10.1016/j.cell.2009.12.023

[41] R. P. Hellens , E. A. Edwards , N. R. Leyland , S. Bean , P. M. Mullineaux , Plant Mol. Biol. 2000, 42, 819. 10890530 10.1023/a:1006496308160

[42] J.‐L. Liu , M.‐M. Chen , W.‐Q. Chen , C.‐M. Liu , Y. He , X.‐F. Song , Plant Physiol. 2022, 188, 1843. 34893900 10.1093/plphys/kiab573 PMC8968415

[43] a) M. Delmans , B. Pollak , J. Haseloff , Plant Cell Physiol. 2017, 58, pcw201; 10.1093/pcp/pcw201 PMC5444569 28100647

[44] N. A. Horst , R. Reski , Plant Methods 2017, 13, 33. 28491120 10.1186/s13007-017-0186-2 PMC5424408

[45] Y. Verhertbruggen , S. E. Marcus , A. Haeger , J. J. Ordaz‐Ortiz , J. P. Knox , Carbohydr. Res. 2009, 344, 1858. 19144326 10.1016/j.carres.2008.11.010

[46] J. Schindelin , I. Arganda‐Carreras , E. Frise , V. Kaynig , M. Longair , T. Pietzsch , S. Preibisch , C. Rueden , S. Saalfeld , B. Schmid , J.‐Y. Tinevez , D. J. White , V. Hartenstein , K. Eliceiri , P. Tomancak , A. Cardona , Nat. Methods 2012, 9, 676. 22743772 10.1038/nmeth.2019 PMC3855844

[47] M. Sraitih , Y. Jabrane , Biomed. Sig. Process. Cont. 2021, 69, 102903.

[48] D. Kim , B. Langmead , S. L. Salzberg , Nat. Methods 2015, 12, 357. 25751142 10.1038/nmeth.3317 PMC4655817

[49] P. Danecek , J. K. Bonfield , J. Liddle , J. Marshall , V. Ohan , M. O. Pollard , A. Whitwham , T. Keane , S. A. McCarthy , R. M. Davies , H. Li , GigaScience 2021, 10, giab008. 33590861 10.1093/gigascience/giab008 PMC7931819

[50] a) M. D. Robinson , D. J. McCarthy , G. K. Smyth , Bioinformatics 2009, 26, 139; 19910308 10.1093/bioinformatics/btp616 PMC2796818

[51] K. Katoh , D. M. Standley , Mol. Biol. Evol. 2013, 30, 772. 23329690 10.1093/molbev/mst010 PMC3603318

[52] S. Capella‐Gutiérrez , J. M. Silla‐Martínez , T. Gabaldón , Bioinformatics 2009, 25, 1972. 19505945 10.1093/bioinformatics/btp348 PMC2712344

[53] A. Stamatakis , Bioinformatics 2014, 30, 1312. 24451623 10.1093/bioinformatics/btu033 PMC3998144

[54] I. Letunic , P. Bork , Nucleic Acids Res. 2021, 49, W293. 33885785 10.1093/nar/gkab301 PMC8265157

[55] C. Chen , H. Chen , Y. Zhang , H. R. Thomas , M. H. Frank , Y. He , R. Xia , Mol. Plant 2020, 13, 1194. 32585190 10.1016/j.molp.2020.06.009

