# Supplementary material for: Morphological Innovation Drives Sperm Release in Bryophytes
Source: Adv Sci (Weinh). 2024 Mar 29;11(20):2306767. doi: 10.1002/advs.202306767 (PMC11132054; doi:10.1002/advs.202306767)
Supplement: Supplementary file 1 — Supporting Information [file ADVS-11-2306767-s005.pdf]

ADVANCED SCIENCE
Open Access

Supporting Information

for *Adv. Sci.*, DOI 10.1002/advs.202306767

Morphological Innovation Drives Sperm Release in Bryophytes

Xinxin Zhang, Ang Bian, Junbo Yang, Ye Liang, Zhe Zhang, Meng Yan, Siqi Yuan and Qun Zhang**

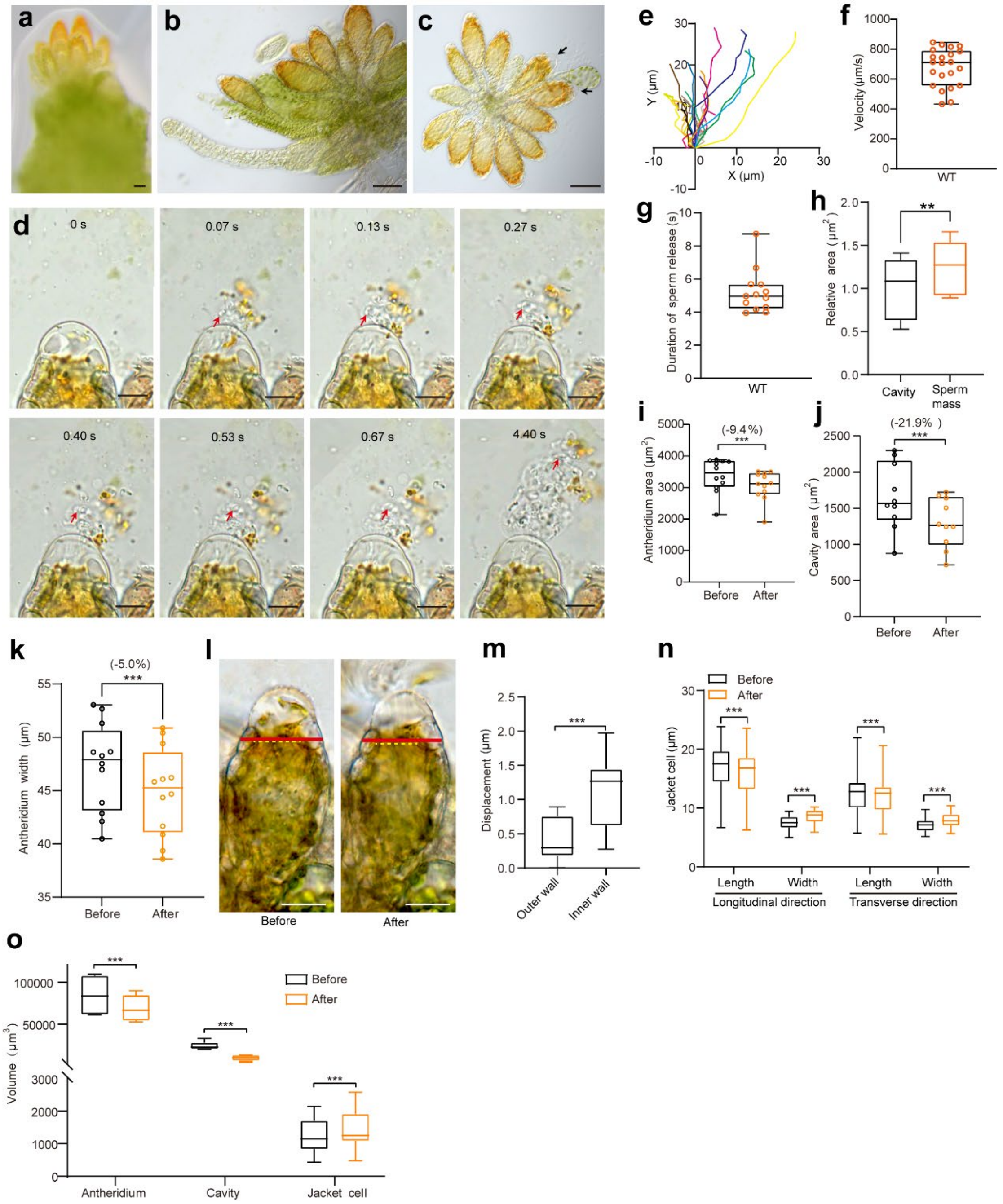

Figure S1. Sperm release in *P. patens*. a) Antheridia formed at the tip of gametophore. b, c) Antheridia. Arrows indicate ruptured antheridium. d) Sperm release from antheridium. Arrows indicate the same sperm cell. e) Sperm cells ejected paths in 1 s; n = 19. f) The ejected speed of the sperm cell at 0.067s. Box plots depict min to max values. Results are mean ± s.d. (n = 22). g) Duration of sperm cell release. The time was calculated from antheridium apex maximum to the cavity empty. Box plots depict min to max values. The result is mean ± s.d. (n = 13). h) Quantification of area of cavity before antheridium burst and released sperm mass after burst. The mean value of cavity

area before burst is set as 1. Box plots depict min to max values. Results are mean ± s.d. (n = 5); **$P < 0.01$; two-tailed paired Student's t-test. i) and j) Quantification of area of the whole antheridium i) and the cavity j) before and after the antheridium burst. Box plots depict min to max values. Results are mean ± s.d. ($n \geq 10$); ***$P < 0.001$; two-tailed paired Student's t-test. k) Quantification of antheridium width before and after the antheridium burst. Box plots depict min to max values. Results are mean ± s.d. (n = 12); ***$P < 0.001$; two-tailed paired Student's t-test. l) Images of antheridium before and after the burst. The red lines indicate the distance between outer walls. The dashed lines indicate the distance between inner walls. m) The displacement of the outer wall and inner wall of jacket cells after the antheridium burst. Box plots depict min to max values. Results are mean ± s.d. (n = 10), ***$P < 0.001$; two-tailed paired Student's t-test. n) The quantification of jacket cell length and width in the longitudinal direction and transverse direction before and after antheridium burst. Box plots depict min to max values. Results are mean ± s.d. ($n \geq 18$); ***$P < 0.001$; two-tailed paired Student's t-test. o) The quantification of volume of antheridium, cavity and jacket cell before and after antheridium burst. Box plots depict min to max values. Results are mean ± s.d. ($n \geq 5$), ***$P < 0.001$; two-tailed paired Student's t-test. Scale bars, 75 µm in a), 50 µm in b, c), 10 µm in d), 20 µm in l).

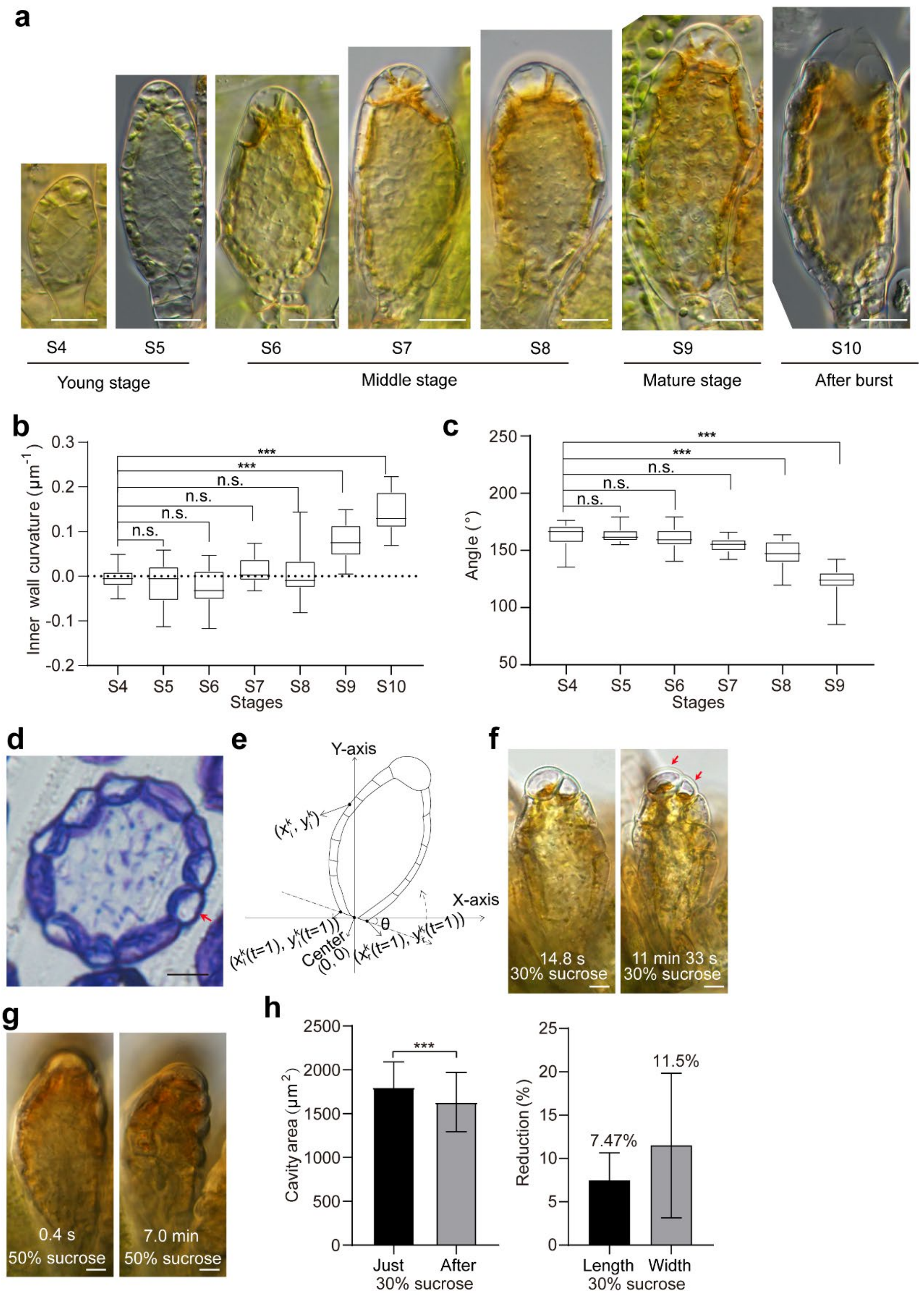

Figure S2. Antheridium development in *P. patens*. a) Images of the antheridium at different developmental stages in the longitudinal direction in *P. patens*. b) Quantification of the inner wall curvature of jacket cells at different stages of antheridium. Box plots depict min to max values. Results are mean ± s.d. ($n \geq 16$); one-

way ANOVA with Dunnett's test; $^{***}P < 0.001$, n.s., no significance ($P > 0.05$). c) Quantification of angles between adjacent inner cell walls at different stages of antheridium. Box plots depict min to max values. Results are mean ± s.d. ($n \geqslant 15$); one-way ANOVA with Dunnett's test; $^{***}P < 0.001$, n.s., no significance ($P > 0.05$). d) The transverse section from the middle region of mature antheridium in *P. patens* with toluidine blue staining. Red arrow indicates highly vacuolated jacket cells. e) The antheridium geometry. f) The mature antheridium treated with 30% sucrose for dehydration. The images were captured from the same antheridium with 30% sucrose treatment for 14.8 s (left panel) and 11 min 33 s (right panel). Arrows indicate apical cells with obvious plasmolysis. g) The mature antheridium treated with 50% sucrose for dehydration. The images were captured from the same antheridium with 50% sucrose treatment for 0.4 s (left panel) and 7.0 min (right panel). Since 50% sucrose caused severe shrinkage of antheridium in a short time, we used 30% sucrose treatment to mature antheridium and then performed subsequent rehydration with pure water. h) The quantification of area, length and width in cavity. They were measured by just adding 30% sucrose solution (for less than 20 seconds) and after 30% sucrose (for more than 9 minutes). The cavity area was measured at just and after 30% sucrose (left panel). The reduction (%) of cavity length and width after adding 30% sucrose for more than 9 minutes (right panel). Results are mean ± s.d. ($n = 10$ for left; $n = 9$ for right); $^{***}P < 0.001$; two-tailed paired Student's *t*-test. Scale bars, 20 μm in a), 10 μm in d, f, g).

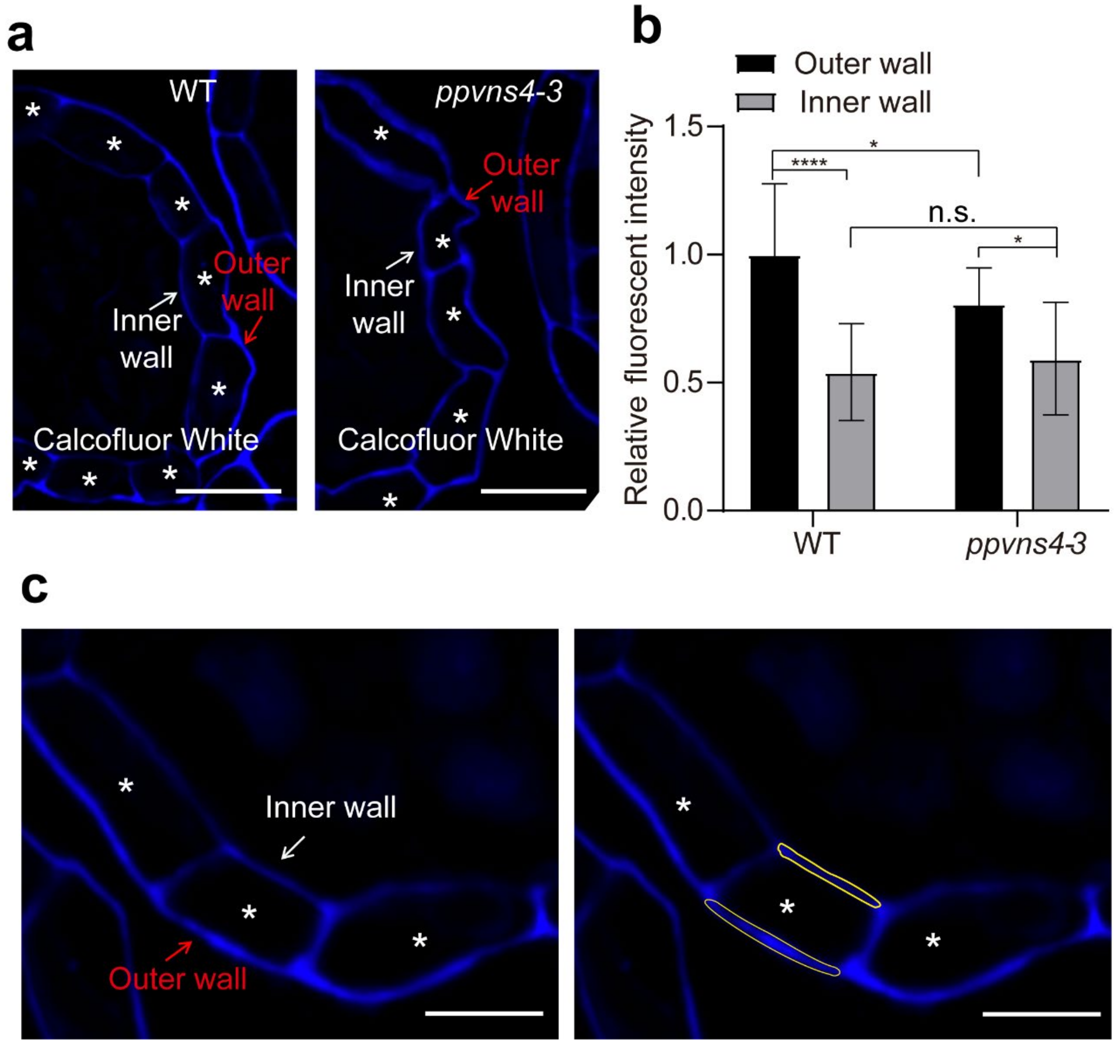

Figure S3. Calcofluor White staining and measurement of fluorescence intensity in the antheridium of *P. patens*. a) Calcofluor White staining (blue signal) of jacket cell walls in wild type and *ppvns4* mutant. The transverse sections of antheridia at S9 stage were stained with Calcofluor White for detecting cellulose. White asterisks indicate jacket cells. b) The relative fluorescent intensity of Calcofluor White staining in jacket cell walls. The mean value of fluorescence intensity in the outer walls of wild type was set as 1. Results are mean ± s.d. ($n \geq 15$); two-way ANOVA with Dunnett's test; $^{****}P < 0.0001$, $^{*}P < 0.05$, n.s., no significance ($P > 0.05$). c) Images showing the representative measurement of fluorescence intensity for Calcofluor White staining in jacket cell walls. The same measurement approach was used for JIM5 and JIM7 immunolabeling. The left panel shows the original image, and the right panel shows how the fluorescence intensity of jacket cell walls was measured in one jacket cell. The yellow lines outline

the selected regions of interest in the outer wall or inner wall in one jacket cell. The average fluorescence intensity in the selected region of a jacket cell was measured. Scale bars, 10 μm in a), 5 μm in c).

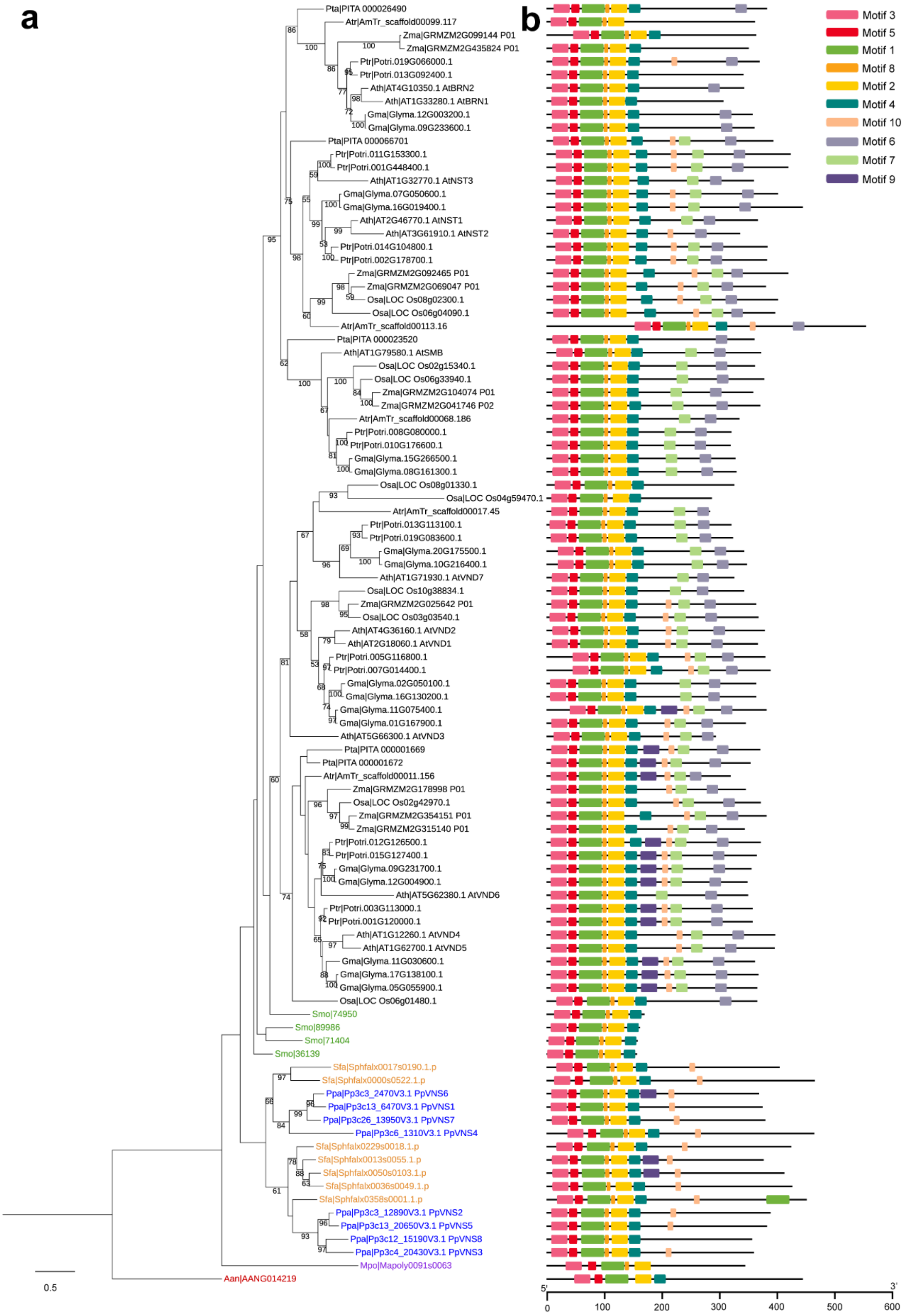

Figure S4. Phylogenetic analysis of VNS family proteins in multiple organisms. a) The

phylogenetic tree of VNS family proteins. The phylogenetic tree was constructed using maximum likelihood analyses and 1000 bootstrap tests, based on the amino acid sequences of the NAC domain from VNS family proteins. The scale bar represents amino acid substitutions per site. Numbers on the nodes indicate the bootstrap values (%). Values below 50% are not shown. Organisms included in the analysis are: Aan (*Anthoceros angustus*), Mpo (*Marchantia polymorpha*), Ppa (*Physcomitrium patens*), Sfa (*Sphagnum fallax*), Smo (*Selaginella moellendorffii*), Atr (*Amborella trichopoda*), Ptr (*Populus trichocarpa*), Ath (*Arabidopsis thaliana*), Osa (*Oryza sativa*), Gma (*Glycine max*), Zma (*Zea mays*), and Pta (*Pinus taeda*). The red color indicates Aan. The purple color indicates Mpo. The blue color indicates Ppa. The orange color indicates Sfa. The green color indicates Smo. b) Schematic representation showing the positions of 10 motifs within the VNS proteins. Different colors indicated different motifs.

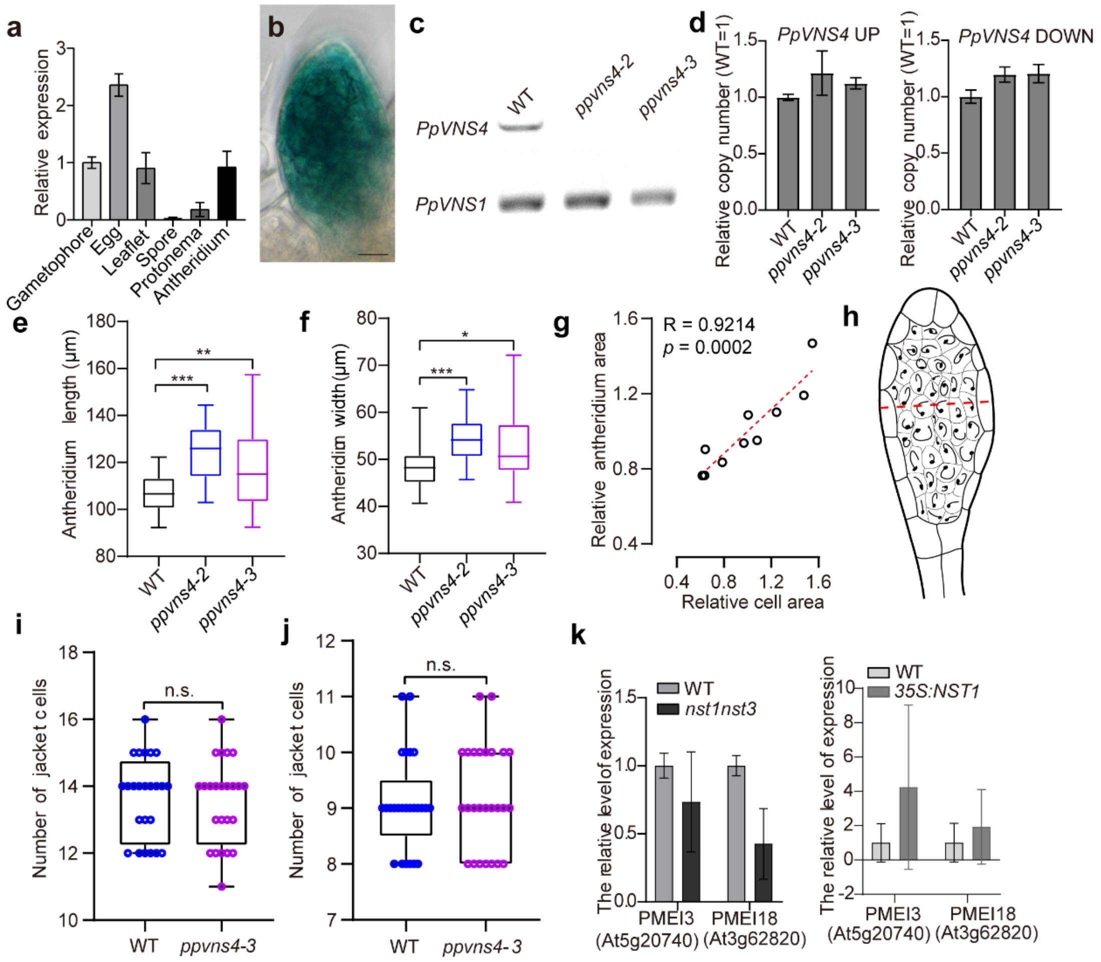

Figure S5. The antheridium of wild type and *ppvns4* mutants in *P. patens*. a) The

relative gene expression analysis of *PpVNS4* in different tissues of *P. patens* using RNA sequencing data. The RPKM (reads per kilobase of transcript per million mapped reads) value of gametophore is set as 1. Results are mean ± s.d. ($n \geq 3$). b) Expression of *PpVNS4-GUS* in antheridium in *proPpVNS4:PpVNS4-GUS* lines. c) Identification of *PpVNS4* knock-out mutants at the genomic level using RT-PCR. The genomic sequence of targeted *PpVNS4* gene was amplified. *PpVNS1* was used as positive control. d) Quantitative PCR analysis was performed to identify the *ppvns4* mutants without unexpected substitutions. Expression of *PpEF1α* served as an internal reference. Results are mean ± s.d. ($n = 3$). e) and f) The length e) and the width f) of antheridium in wide type and *ppvns4* mutants. Box plots depict min to max values. Results are mean ± s.d. ($n \geq 20$); one-way ANOVA with Dunnett's test; *$P < 0.05$; **$P < 0.01$; ***$P < 0.001$. g) Correlation between relative jacket cell area and antheridium area. Relative antheridium and jacket cell areas in the wild type and *ppvns4* were used for linear regression analysis. h) An antheridium diagram. Red dashed line indicates the middle region for counting jacket cell numbers in a transverse section. i) and j) The number of jacket cells in the longitudinal i) and transverse section j). Box plots depict min to max values. Results are mean ± s.d. (n ≥ 24); Welch's *t*-test; n.s.: no significance ($P > 0.05$). k) The relative gene expression analysis of *PMEI3* and *PMEI18* from microarray data. The expression level in wild type is set as 1. Results are mean ± s.d. ($n = 3$ (left panel), 4 (right panel)); Welch's *t*-test. Scale bars, 10 μm in b).

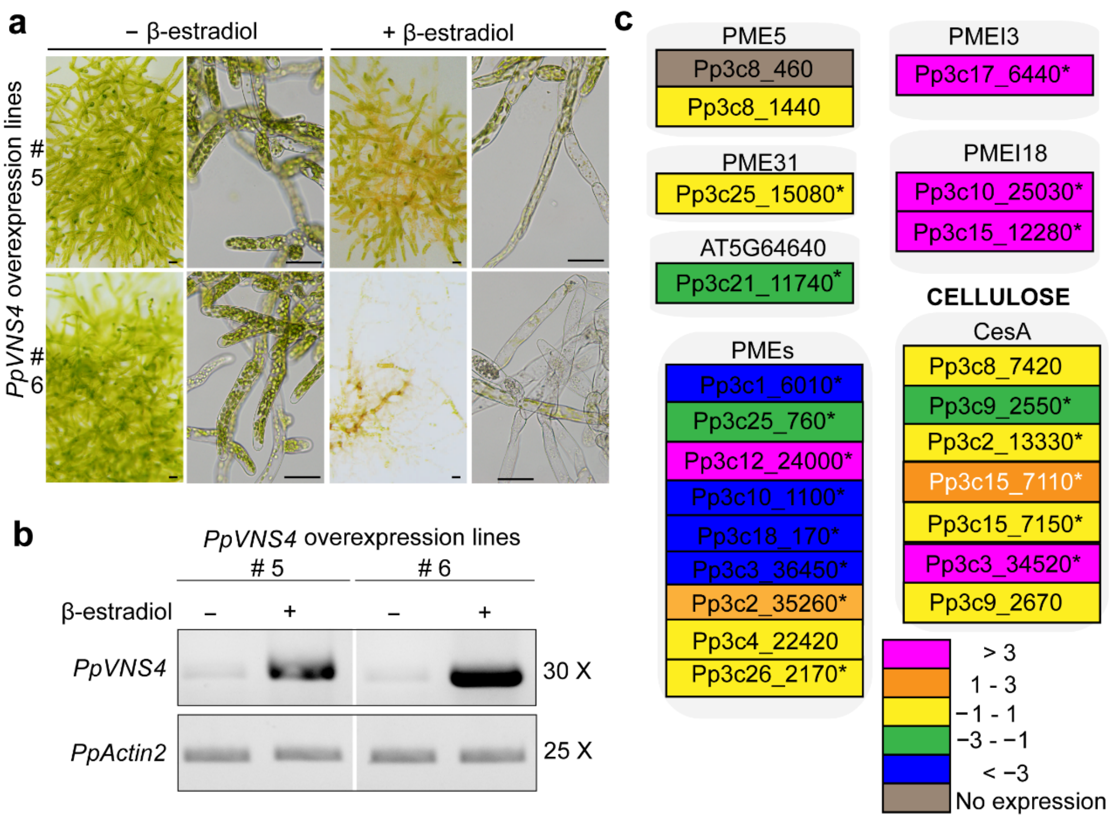

Figure S6. Changed expression of putative cell wall-related genes by overexpression of *PpVNS4* gene in wild type of *P. patens*. a) Four-day protonemata of *PpVNS4* overexpression lines were treated with or without 1 μM β-estradiol for 18 h. b) Semi-qRT-PCR of β-estrogen-inducible *PpVNS4* overexpression in *P. patens* in a). The *PpActin2* gene was used as the internal control. The PCR cycle numbers are indicated on the right. The overexpression line 6 was used for further analysis. c) Changed expression profiles of *P. patens* genes putatively homologous to well-characterized *A. thaliana* genes associated with cell wall biosynthesis. The color scale represents the log2 fold changes of gene expression. Asterisks indicate significant differences. Scale bar, 100 μm in a).

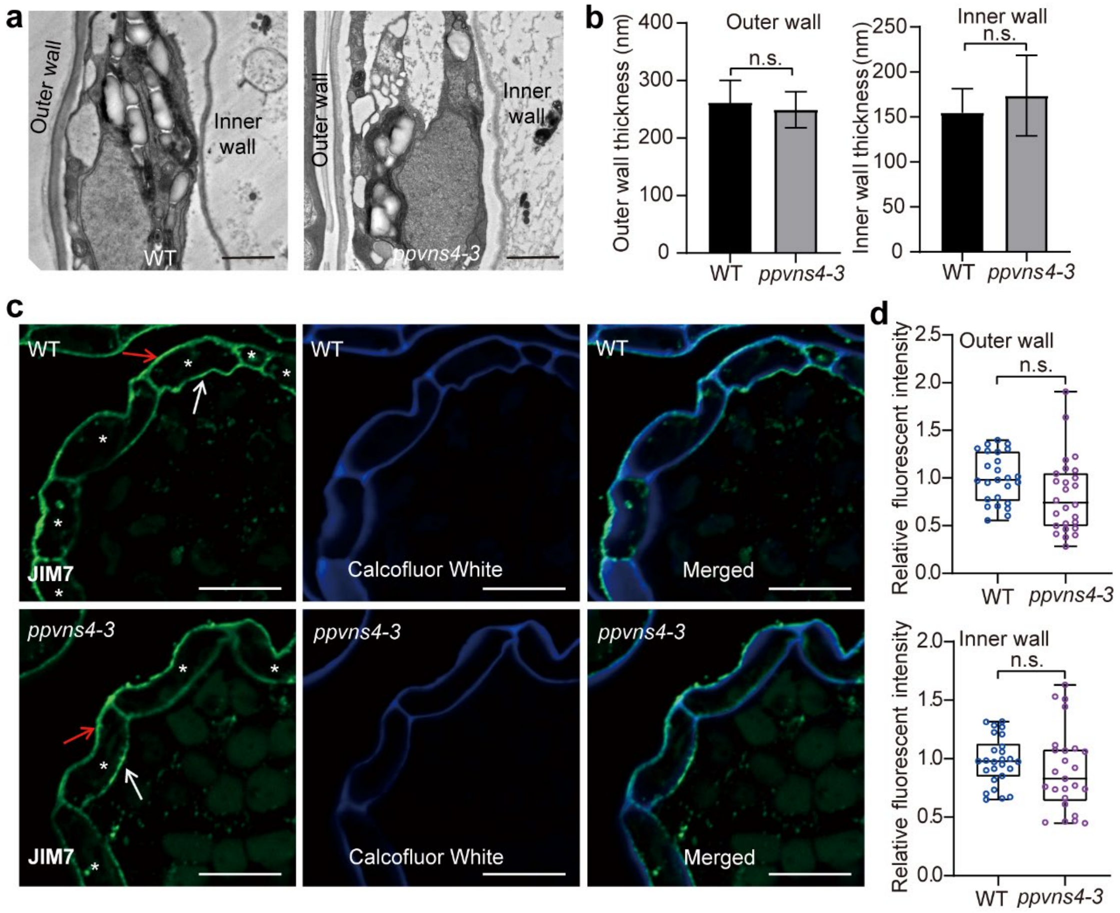

Figure S7. The thickness and highly methylesterified pectin contents in jacket cell walls of antheridium in *P. patens* wild type and *ppvns4* mutants. a) TEM images of jacket cells in the longitudinal section of mature antheridium in wild type and *ppvns4*. b) The quantification of thickness of jacket cell walls. Results are mean ± s.d. ($n \geq 20$); n.s., no significance ($P > 0.05$); Welch's *t*-test. c) Labeling of transverse sections of antheridium with JIM7 (green signal) for highly methylesterified pectin epitope and cellulose-binding Calcofluor White (blue signal) at S8 stage. White asterisks indicate jacket cells. Red arrows indicate the outer cell wall. White arrows indicate the inner cell wall. d) Quantification of relative fluorescent intensity detected by antibody JIM7 for outer cell walls and inner cell walls of jacket cells. The mean value of fluorescence intensity of wild type is set as 1. Box plots depict min to max values. Results are mean ± s.d. ($n \geq 25$); Welch's *t*-test; n.s., no significance ($P > 0.05$). Scale bars, 2 µm in a), 10 µm in c).

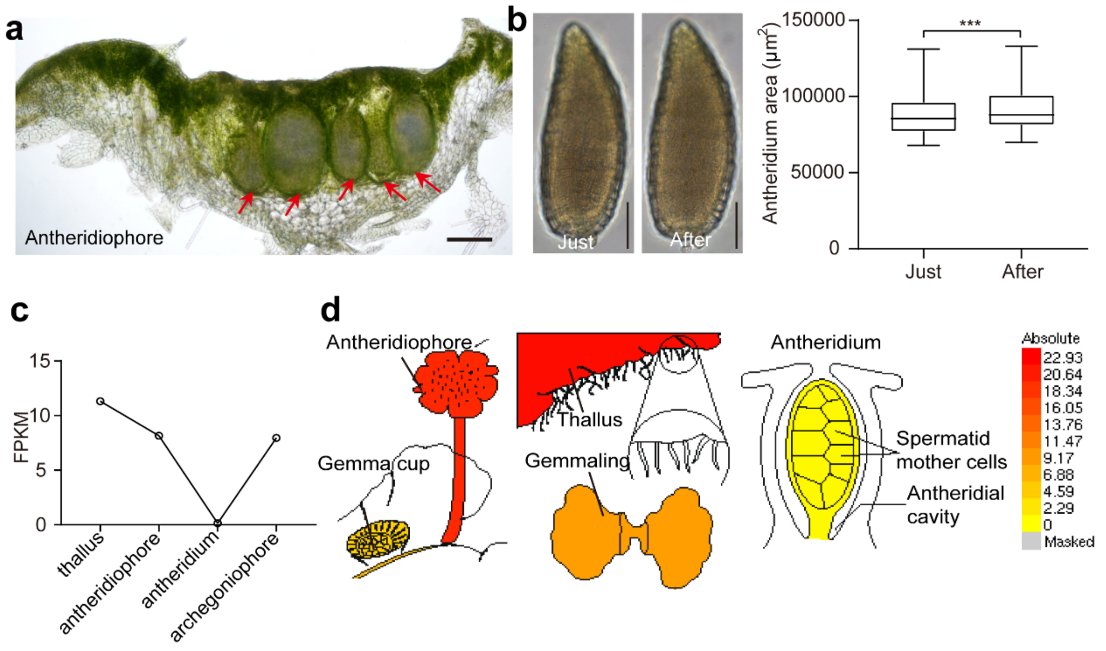

Figure S8. Mp*VNS* is not expressed in the antheridia of *M. polymorpha*. a) Longitudinal section of antheridiophore in *M. polymorpha*. Red arrows indicate the antheridia. b) the antheridium size of *M. polymorpha* increased after adding water. The images of the same antheridium of *M. polymorpha* just adding water and after adding water (left panel). Quantification of antheridium of *M. polymorpha* just and after adding water (right panel). Box plots depict min to max values. Results are mean ± s.d. ($n \geq 25$); two-tailed paired Student's *t*-test; $***P < 0.001$. c) RNA sequencing data for expression of Mp*VNS* gene during *M. polymorpha* growth were analyzed across different tissues. d) Expression patterns of Mp*VNS* gene in different tissue of *M. polymorpha* in eFP browser. Mp*VNS* expression levels (absolute values) based on a transcriptomic data set. Scale bars, 200 μm in a), 100 μm in b).

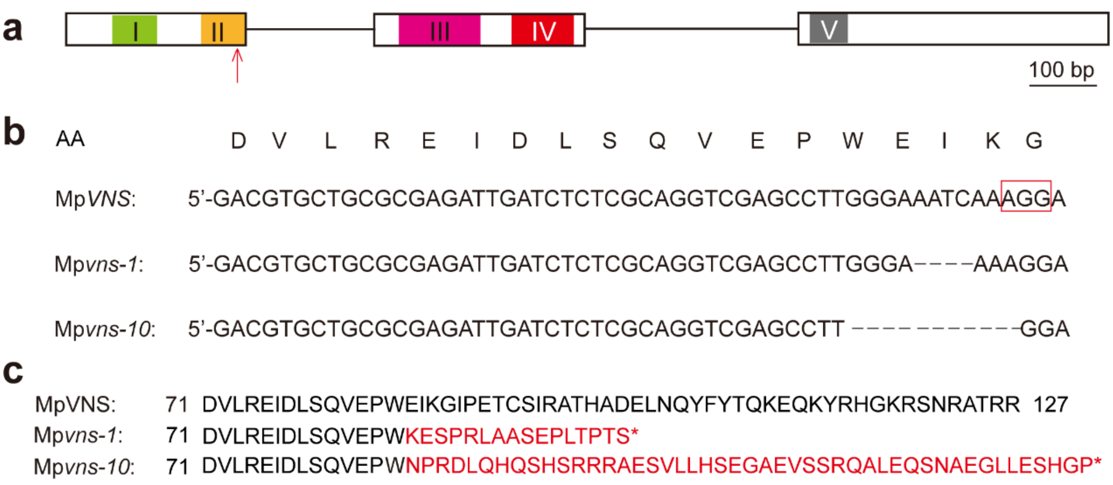

Figure S9. Identification of Mp*vns* mutants *in M. polymorpha*. a) Mp*VNS* gene structure. Boxes and lines indicate exons and introns, respectively. Colored boxes indicate NAC subdomains. Red arrow indicates the targeting position of guide RNA. b) Mp*vns* CRISPR mutants. AA indicates the putative amino acid (AA) sequence. The box in red indicates protospacer adjacent motif (PAM) sequence. Dashed lines indicate deletions, with 4 bp deletion in Mp*vns-1* and 11 bp deletion in Mp*vns-10*, respectively. c) Genotyping of Mp*vns* alleles. Asterisks represent the translation termination.

Movie S1. Sperm release from antheridium in the moss *P. patens*. Scale bar: 10 µm.

Movie S2. Released sperm cells from antheridium of *P. patens* rotate aimlessly in irregular circles in sperm mass. Scale bar: 5 µm.

Movie S3. Released sperm cell from antheridium of *P. patens* freely swims by vibrating its long flagella. Scale bar: 5 µm.

Movie S4. Antheridium burst in the moss *P. patens*. Scale bar: 10 µm.

Movie S5. Sperm cannot be released from isolated antheridium of *M. Polymorpha* in water. Scale bar: 100 µm.

Table S1. List of primers used in this study.

Table S2. List of putative *P. patens* homologous of *A. thaliana* genes related to *PME* and *PMEI*.

Table S3. RNA-seq data for transgenic *P. patens* by induction of *PpVNS4* overexpression with or without 1 µM β-estradiol for 18 hours.
